# Decreased miR-128-3p in serum exosomes from polycystic ovary syndrome induces ferroptosis in granulosa cells via the p38/JNK/SLC7A11 axis through targeting *CSF1*

**DOI:** 10.1038/s41420-025-02331-0

**Published:** 2025-02-18

**Authors:** Yanqiu Lv, Shengzhong Han, Fuliang Sun, Yuyang Zhang, Xinglin Qu, Hao Li, Weiyu Gu, Qinglong Xu, Shunfa Yao, Xuan Chen, Yi Jin

**Affiliations:** 1 Department of Animal Science, College of Agriculture, Yanbian University, Jilin, China; 2 Jilin Provincial Key Laboratory of Transgenic Animal and Embryo Engineering, Yanbian University, Yanji, China

**Keywords:** miRNAs, Diagnostic markers

## Abstract

Increasing evidence suggests that non-coding small RNAs (miRNAs) carried by exosomes (EXOs) play important roles in the development and treatment of polycystic ovary syndrome (PCOS). In this study, we demonstrate that PCOS mouse serum-derived EXOs promote granulosa cells (GCs) ferroptosis, and induce the occurrence of a PCOS-like phenotype in vivo. Notably, EXO miRNA sequencing combined with in vitro gain- and loss-of-function assays revealed that miR-128-3p, which is absent in the serum-derived EXOs of mice with PCOS, regulates lipid peroxidation and GC sensitivity to ferroptosis inducers. Mechanistically, overexpression of *CSF1*, a direct target of miR-128-3p, reversed the anti-ferroptotic effect of miR-128-3p. Conversely, ferroptosis induction was mitigated in *CSF1*-downregulated GCs. Furthermore, we demonstrated that miR-128-3p inhibition activates the p38/JNK pathway via *CSF1*, leading to NRF2-mediated down-regulation of *SLC7A11* transcription, which triggers GC iron overload. Moreover, intrathecal miR-128-3p AgomiR injection into mouse ovaries ameliorated PCOS-like characteristics and restored fertility in letrozole-induced mice. The study reveals the pathological mechanisms of PCOS based on circulating EXOs and provides the first evidence of the roles of miR-128-3p and *CSF1* in ovarian GCs. This discovery is expected to provide promising therapeutic targets for the treatment of PCOS.

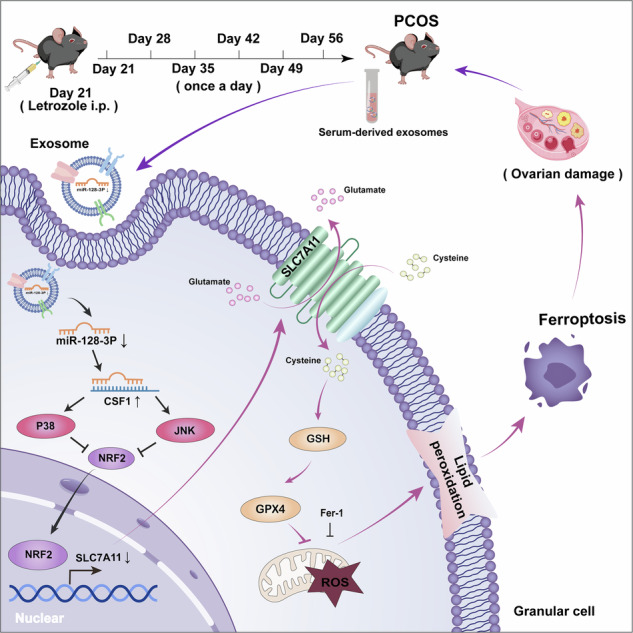

## Highlights

- Serum exosomes from PCOS mice induce GC ferroptosis.
- Serum exosomes of PCOS-like mice have lower miR-128-3p levels.
- Exosome effects are mediated by the miR-128-3p-*CSF1*-p38/JNK signalling axis.
- miR-128-3p and *CSF1* are regulators mediating ferroptosis in ovarian GCs.
- miR-128-3p is a novel potential biomarker for the diagnosis and treatment of PCOS.

## Introduction

Polycystic ovary syndrome (PCOS) is a prevalent endocrine and metabolic disorder condition that affects 6–20% of females in their reproductive years [1]. The clinical manifestations of PCOS are diverse and include hirsutism, amenorrhoea, hyperinsulinaemia, obesity, hyperandrogenaemia, and the presence of polycystic ovaries identified through ultrasonography [2, 3]. Ovarian granulosa cells (GCs), which constitute the largest cell population within ovarian follicles, play a pivotal role in the follicular microenvironment [4]. Metabolic perturbations and dysfunctional regulated cell death are closely associated with ovarian dysfunction [5, 6]. Recent studies have demonstrated an association between the pathogenesis of PCOS and oxidative stress-induced cell death in GCs [4, 7]. However, the precise mechanisms underlying GC death remain elusive.

Exosomes (EXOs), small extracellular vesicles secreted by most cell types in the body, are present in all types of body fluids, including serum, and play a critical role in cell-cell communication by selectively packaging and transporting bioactive materials such as proteins, lipids, mRNA, and miRNA [8, 9]. Significantly, miRNAs enclosed within EXOs are protected from RNase degradation, enhancing their potential as clinical biomarkers and prognostic targets in a wide array of diseases, including diseases affecting the reproductive system [10], muscle-wasting disorders [11], and cancer [12]. Recent research has seen a growing focus on the role of EXOs in PCOS, with their findings highlighting the potential regulatory effects of EXOs and their miRNAs on follicular development in patients with PCOS [13, 14]. Nevertheless, research on PCOS-associated EXO miRNAs is still in its early stages.

Ferroptosis, a recently identified form of programmed cell death, is characterised by an imbalance in the intracellular redox system, which results in cell membrane lipid peroxidation and eventual cell death [15]. Two primary pathways contribute to the development of ferroptosis, the enzyme-regulated pathway, which activates ferroptosis by inhibiting glutathione peroxidase 4 (GPX4) and promoting lipid peroxidation [16], and the transporter-dependent pathway, which activates ferroptosis by inhibiting solute carrier family 7 member 11 (SLC7A11) [17]. The role of ferroptosis in PCOS is a novel finding, with its occurrence being accompanied by an imbalance in the REDOX system in GCs [18]. Recent research underscores the growing significance of ferroptosis in PCOS [19–21]. Importantly, studies have shown that inhibiting ferroptosis in the ovaries can alleviate PCOS symptoms and slow disease progression [18]. Thus, elucidating the mechanisms underlying ferroptosis in the ovaries will significantly enhance our understanding of PCOS pathogenesis and provide new insights for developing therapeutic strategies.

This study utilized a mouse model of PCOS to explore the mechanistic role of serum-derived EXOs in GC ferroptosis. Here, we demonstrate that the serum-derived EXOs of mice with PCOS promote GC lipid peroxidation-induced ferroptosis, thereby impairing ovarian follicle development, by delivering EXOs lacking miR-128-3p. Specifically, the upregulation of *CSF1*, a target gene of miR-128-3p, is a major trigger for GC ferroptosis, which repressing NRF2 expression by activating the p38/JNK signalling pathway inhibits *SLC7A11* transcription. Furthermore, we demonstrated that miR-128-3p AgomiR exerts significant beneficial effects on follicular development in PCOS mice, consequently increasing litter size. This study sheds light on the underlying mechanism and progression of PCOS, providing new promising therapeutic targets and potential biomarkers for the disease.

## Results

### PCOS serum-derived EXOs promote GC ferroptosis

**Fig. 1:**
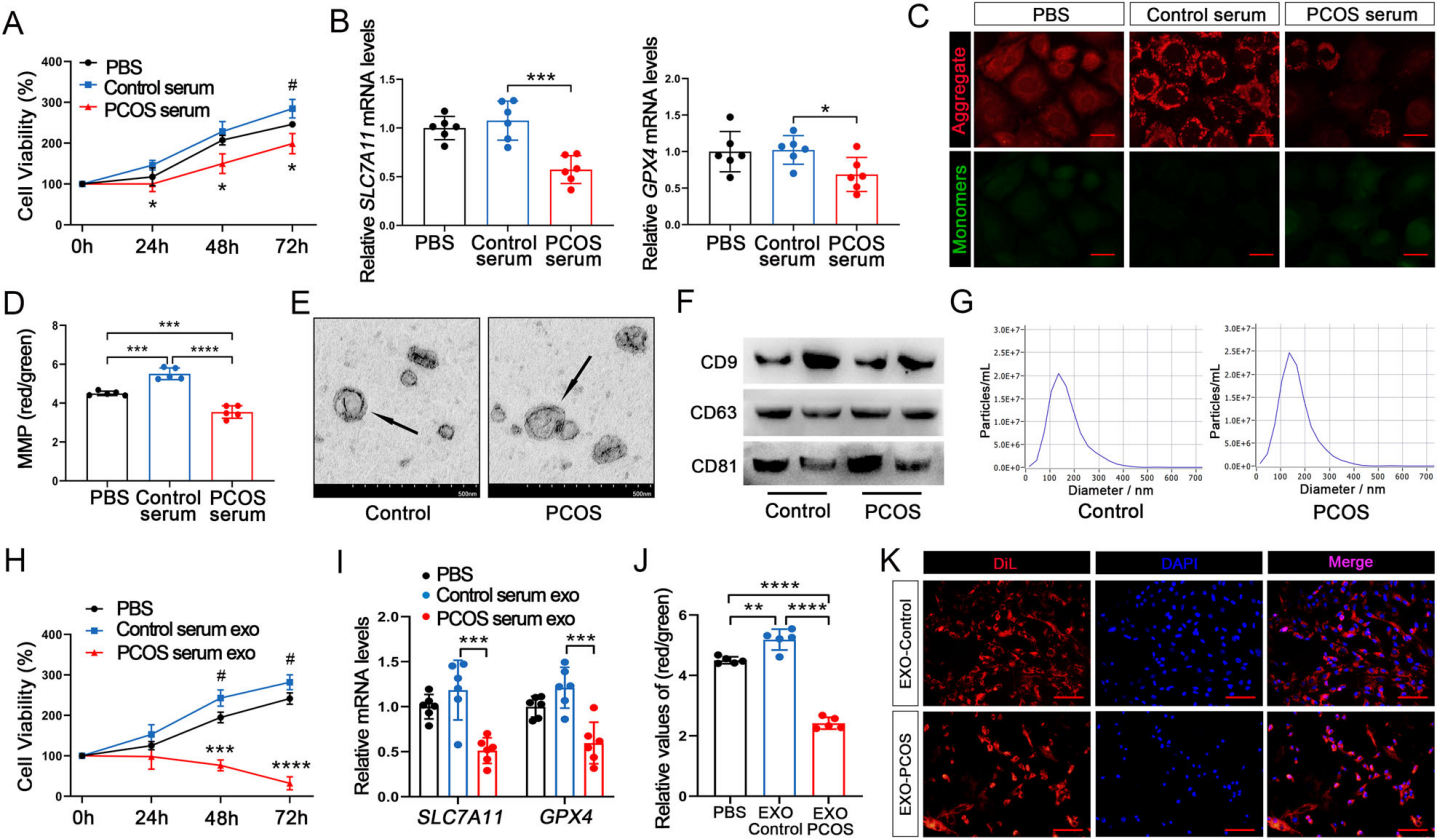
PCOS mouse serum and serum-derived exosomes (EXOs) promote granulosa cell (GCs) ferroptosis. **A** Cell viability of GCs incubated with control and PCOS mouse serum for 0, 24, 48, or 72 h. *P < 0.05 vs Control serum, # *P* < 0.05 vs Control serum. **B** mRNA expression levels of ferroptosis-related genes in GCs as determined using qRT-PCR. **C**, **D** Determination of mitochondrial membrane potential (MMP) levels in GCs. Scale bar: 30 μm. **E** Transmission electron microscopic (TEM) analysis of serum-derived EXOs. Scale bar: 500 nm. **F** Western blot analysis of the EXO-specific marker proteins, CD9, CD63, and CD81. **G** EXO particle size as determined through the ZetaView analysis. **H** Cell viability of GCs incubated with serum EXOs for 0, 24, 48, or 72 h. ***P < 0.001 vs Control serum exo, ****P < 0.0001 vs Control serum exo, # *P* < 0.05 vs Control serum exo. **I** Relative mRNA levels of *SLC7A11* and *GPX4* in serum EXO-treated GCs. **J** MMP levels in GCs treated with EXOs as determined using JC-1 staining. **K** Co-culture assay showing that labelled serum EXOs can enter GCs. Scale bar: 200 μm. Data are presented as Mean ± SD. *P < 0.05, **P < 0.01, ***P < 0.001, ****P < 0.0001.

The role of circulating factors in the regulation of tissue homeostasis and disease progression has been well established [22, 23]. Hence, we hypothesised that information stored within circulating serum in individuals with PCOS contains pathogenic signals that induce ovarian GC dysfunction. Consistent with our observations in the ovaries of PCOS mice, GCs showed decreased cell viability (Fig. 1A). Recent research highlights a significant decrease in the ferroptosis regulator GPX4 in PCOS patients, alongside abnormal mitochondrial structures in GCs [20]. Similarly, treatment with PCOS mice serum suppressed the expression of ferroptosis-related genes, including *SLC7A11* and *GPX4* (Fig. 1B). Given the association between ferroptosis and mitochondrial dysfunction based on membrane impairment due to lipid peroxidation [24, 25], we assessed the mitochondrial membrane potential (MMP) in serum-treated cells using JC-1 staining. As expected, after serum treatment, MMP in GCs decreased (Fig. 1C, D). These results indicated that PCOS mouse serum may contain functional factors that promote GC ferroptosis.

The effect of serum-derived factors on recipient cells is mediated by the transportation of membrane-enclosed paracrine factors and EXOs [22, 26]. To determine whether EXOs mediate the observed effects of PCOS mouse serum on GC ferroptosis, we isolated mouse serum EXOs and observed their morphological characteristics using Transmission electron microscopic (TEM) (Fig. 1E). And EXO signature protein (CD9, CD63, and CD91) expression was high in EXO lysates (Fig. 1F). The size of separated particles was in the range of 30–300 nm (Fig. 1G), which was consistent with those typically found upon exosome characterisation. Then, we incubated the isolated EXOs with GCs. Surprisingly, treatment with PCOS serum-derived EXOs induced phenotypes similar to those induced by treatment with serum (Fig. 1H–J). Labelled EXOs were observed in GCs and emitted a significant red fluorescence, confirming GC uptake of serum-derived exosomes (Fig. 1K).

### PCOS mouse serum-derived EXOs induce oestrous disorders and increase ferroptosis in pubertal mice

**Fig. 2:**
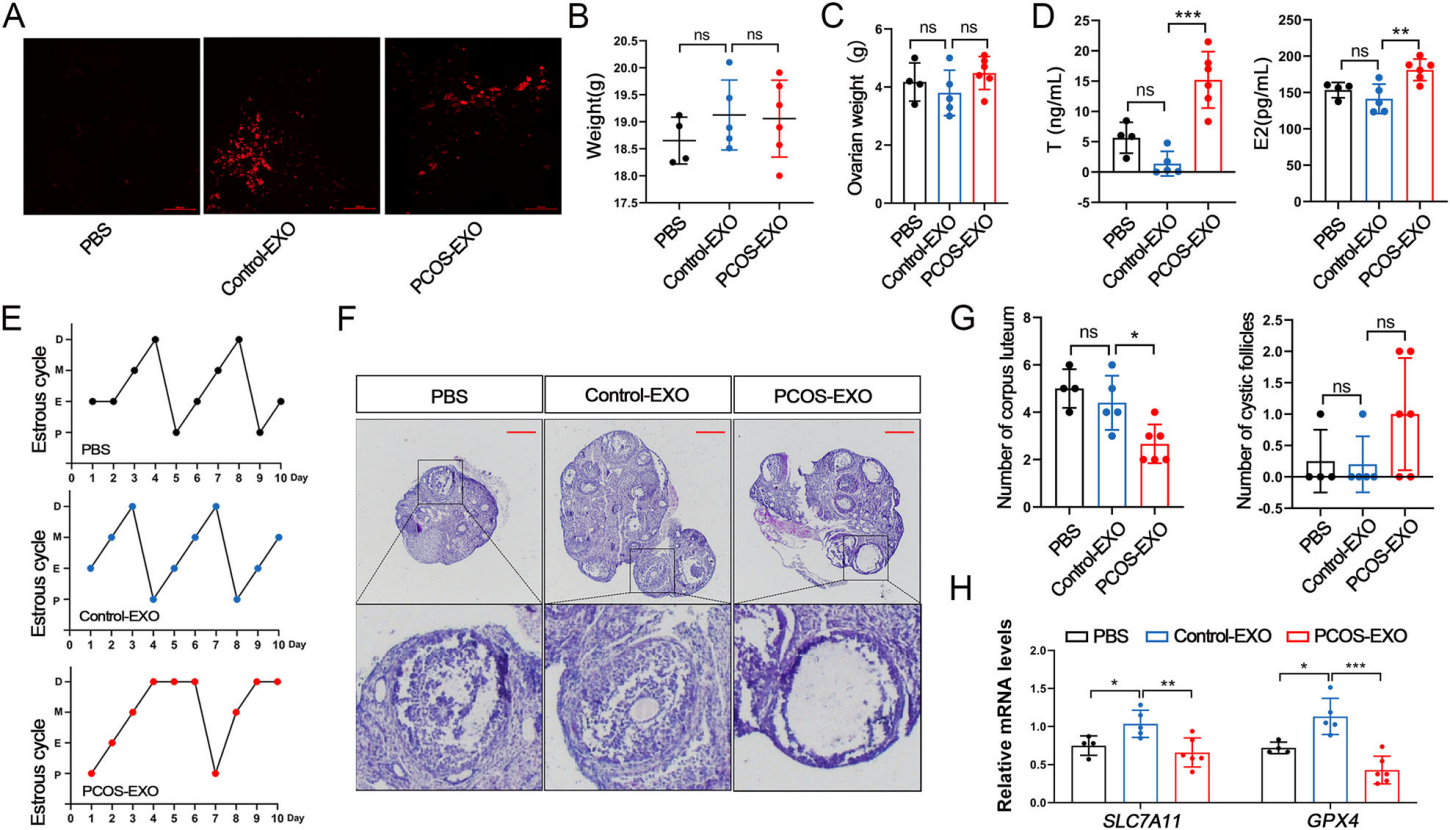
PCOS mouse serum-derived EXOs induce oestrous disorders and increase ferroptosis in mice. **A** Immunofluorescence imaging of ovarian tissues 24 h after serum-derived EXO injection, Scale bar: 100 μm. **B**, **C** Measurement of body and ovarian weights in mice in each group. **D** Steroid sex hormone concentrations in the sera of mice from the different groups as determined via ELISA. **E** Evaluation of representative oestrous cycles after injection of serum EXOs, “P:” proestrous, “E:” oestrous, “M:” metoestrous, “D:” dioestrus. **F**, **G** Corpus luteum and cystic follicle counts following H&E staining. Scale bar: 300 μm. **H** Ferroptosis-related gene mRNA expression levels. Data are presented as Mean ± SD. Ns *P* > 0.05, *P < 0.05, **P < 0.01, ***P < 0.001, ****P < 0.0001.

To further investigate the role of PCOS mouse serum-derived EXOs, we conducted in vivo experiments. Visible DiL fluorescence was observed in mouse ovarian tissues within 48 h of EXO injection (Fig. 2A). In addition, no significant changes in body and ovarian weight were observed within two weeks of PCOS mouse serum-derived EXO injection (Fig. 2B, C); however, serum E2 and T levels significantly increased (Fig. 2D). Steroid hormones play a crucial regulatory role in the oestrous cycle. As expected, we observed oestrous cycle dysregulation in mice two weeks after PCOS mouse serum-derived EXO injection (Fig. 2E). Histological analysis of ovarian slices stained with HE showed a significant decrease in corpus luteum counts in mouse ovaries following PCOS mouse serum-derived EXO injection (Fig. 2F, G). In addition, consistent with the in in vitro findings, PCOS serum-derived EXO injection significantly suppressed *SLC7A11* and *GPX4* expression (Fig. 2H). In summary, these outcomes indicated that PCOS mouse serum-derived EXOs mediate ferroptosis in ovarian GCs.

### PCOS mouse serum-derived EXOs exhibit low miR-128-3p expression

**Fig. 3:**
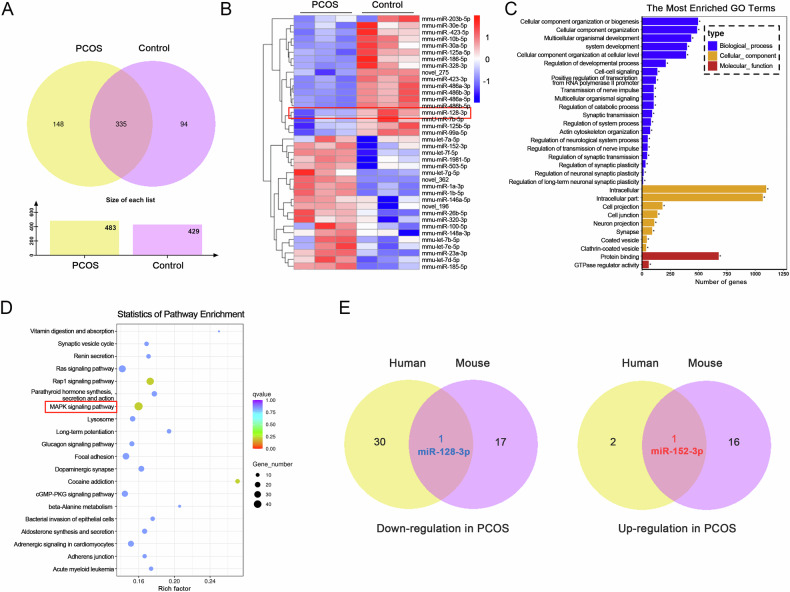
miRNA expression profiles of serum-derived EXOs from PCOS and control mice. **A** Venn diagram showing common and specific miRNA expression in control and PCOS mouse serum-derived EXOs. **B** Hierarchical clustering analysis showing 18 downregulated and 17 upregulated miRNAs, as well as significantly decreased miR-128-3p expression, in PCOS mouse serum-derived EXOs. **C** GO and (**D**) KEGG enrichment analyses of differentially expressed miRNAs. **E** miRNAs that are co-downregulated and co-upregulated in humans and mice in the database. Serum samples from 250 mice in each group were pooled for EXO isolation. The extracted EXO were subjected to miRNA-seq with three technical replicates performed per group to ensure reliability. Data are presented as Mean ± SD. *P < 0.05, **P < 0.01, ***P < 0.001.

miRNAs are important modulators of intricate genetic and epigenetic gene and/or protein regulation [27]. EXOs exhibit a relatively higher miRNA content than other components, such as DNA, proteins, lipids, and mRNA [8]. Therefore, we conducted a comprehensive analysis of miRNA expression in PCOS mouse serum-derived EXOs using high-throughput sequencing. A total of 577 miRNAs were identified, with 335 of them exhibiting co-expression in both groups. Specifically, 94 and 148 miRNAs were exclusively expressed in control and PCOS mice, respectively (Fig. 3A). Total 35 differentially expressed miRNAs were identified, comprising 17 upregulated and 18 downregulated miRNAs in PCOS mice (Fig. 3B). GO functional enrichment analysis revealed that the target genes participate in diverse biological processes, especially cellular component organisation or biogenesis, multicellular organismal development, system development, and cell–cell signalling (Fig. 3C). Additionally, KEGG enrichment analysis revealed the involvement of these target genes in pivotal signalling pathways, such as the MAPK, Ras, and Rap1 pathways, which regulate cellular proliferation, differentiation, and ferroptosis (Fig. 3D) [4, 28–30].

Although we observed altered miRNA expression profiles in PCOS mouse serum-derived EXOs, it is unclear which miRNAs mediate the effects of EXOs on GC ferroptosis. To further screen differential miRNAs that may regulate PCOS and serve as potential biomarkers, we correlated our sequencing results with the publicly available RNA-seq dataset (GSE142819) of plasma exosomes from 15 PCOS patients and 15 controls. The results showed that only miR-128-3p was co-downregulated while miR-152-3p was co-upregulated in PCOS patients and PCOS-like mice (Fig. 3E). Importantly, when the sample size was increased to 150 (75 PCOS patients and 75 controls), the difference in expression of these two miRNAs remained [31]. Independent studies by Ding et al. and Song et al. have reported a significant reduction in the expression of miR-128, which shares the same sequence as miR-128-3p, in patients with PCOS [32, 33]. These consistent findings prompted us to investigate further the potential roles and mechanisms of these miRNAs in the development of PCOS.

### miR-128-3p inhibits GC ferroptosis

**Fig. 4:**
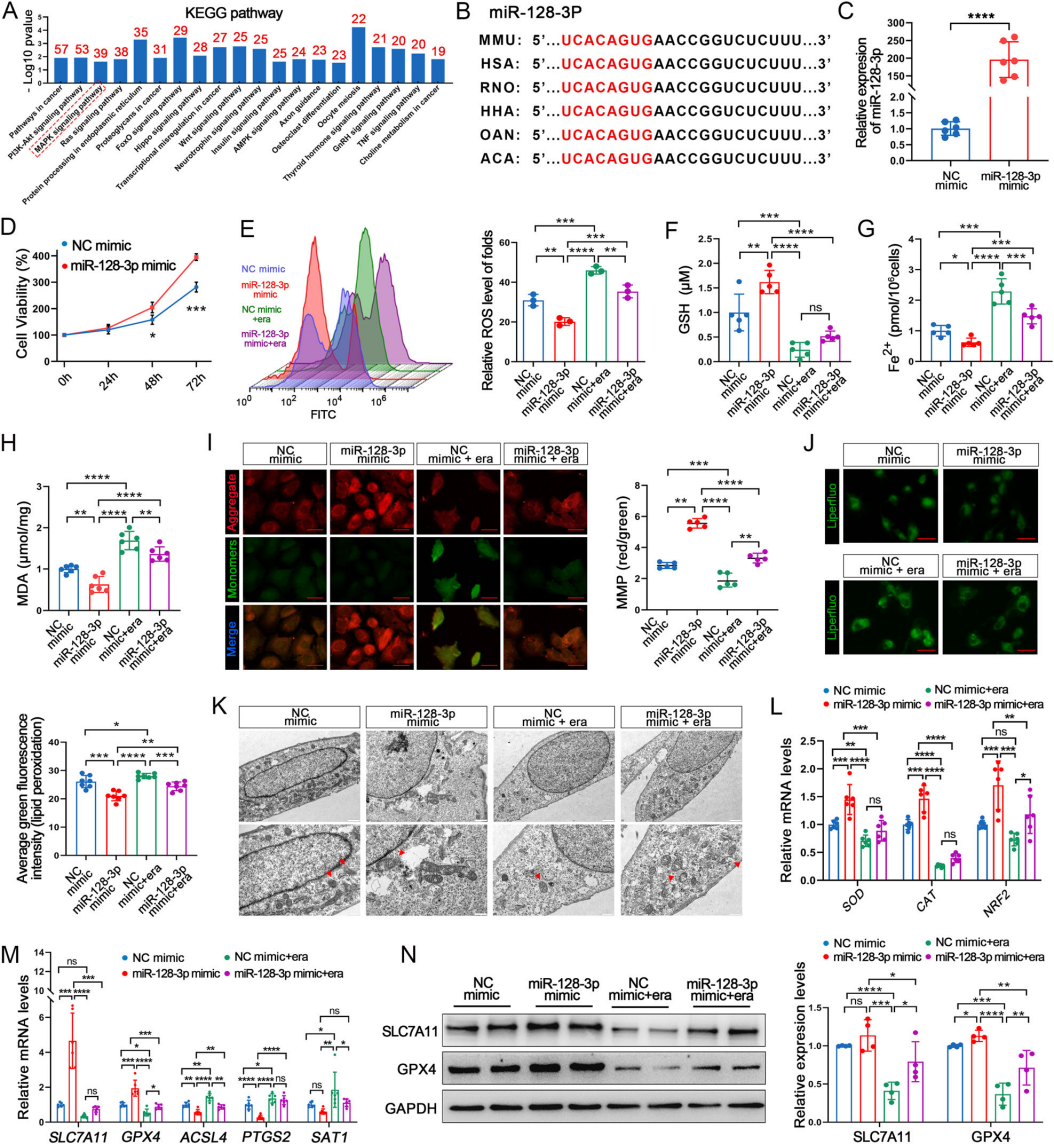
Overexpression of miR-128-3p inhibits GC ferroptosis. **A** Analysis of signalling pathways involving miR-128-3p using Diana Tools, the red numbers indicate the number of target genes enriched in this pathway. **B** Sequence of mature miR-128-3p for different species. **C** miR-128-3p mimic treatment increased miR-128-3p levels in GCs. **D** GC cell viability as determined through the CCK-8 assay. **E** ROS, (**F**) GSH, (**G**) Fe^2+^ and (**H**) MDA levels in GCs treated with erastin and the miR-128-3p mimic. **I** Measurement of MMP levels in GCs treated with erastin and the miR-128-3p mimic. Scale bar: 30 μm. **J** Lipid Peroxidation Levels Assessed by Liperfluo Staining. Scale bar: 30 μm. **K** Low- and high-magnification images obtained by TEM, The red arrows indicate the outer mitochondrial membrane was ruptured and the mitochondrial cristae decreased or disappeared. Scale bar: 1 μm and 500 nm. **L** Relative mRNA levels for oxidative stress-related genes in GCs. **M**, **N** mRNA and protein levels for ferroptosis-related genes. Data are presented as Mean ± SD. Ns *P* > 0.05, *P < 0.05, **P < 0.01, ***P < 0.001, ****P < 0.0001.

miR-128-3p has been reported to be a pro-proliferative factor in the GCs of young females with diminished ovarian reserves [34]. Therefore, we choose miR-128-3p as candidate miRNA mediated ovarian follicle development. Using Diana Tools to perform enrichment analysis of the signalling pathways involving miR-128 target genes revealed that these pathways include the PI3K-Akt, MAPK, and Ras pathways (Fig. 4A), with the MAPK signalling pathway being a key ferroptosis pathway [30]. In addition, the conservation of the miR-128-3p seed sequence in humans and mice suggests its functional consistency across different species (Fig. 4B). Therefore, we focused on determining the exact role of miR-128-3p in mGCs and found that transfecting mouse ovarian GCs with miR-128-3p mimics resulted in a 200-fold upregulation in miR-128-3p expression and increased in cell viability (Fig. 4C, D). Era, an inhibitor of the system Xc-, was introduced to induce ferroptosis in granulosa cells. A key feature of ferroptosis is the accumulation of reactive oxygen species (ROS), typically triggered by the depletion of GSH. Impairment of mitochondrial function was observed in the PCOS patients, evident in a decrease in oxygen consumption, an increase in reactive oxygen species production, a decrease in the GSH/GSSG ratio and GSH levels, and an undermining of the membrane potential [35, 36]. Therefore, we assessed the ROS and GSH levels, to determine whether miR-128-3p could be a potential target for ferroptosis in GCs. Interestingly, miR-128-3p overexpression significantly inhibited Era-induced ROS production and GSH depletion (Fig. 4E, F). After assessing the content of Fe^2+^ MDA, lipid peroxidation, and alongside MMP levels, we discovered that the miR-128-3p mimic significantly improved the MDA, lipid peroxidation, and Fe^2+^ production and mitigated the decrease in MMP caused by Era (Fig. 4G–J). In addition, electron microscopy analysis revealed distinctive ferroptosis-related changes in GCs from patients with PCOS, including reduced mitochondrial volume, disrupted outer membranes and decreased or absent cristae [20, 37]. In this study, the TEM demonstrated that miR-128-3p overexpression significantly alleviated these changes. Specifically, it restored mitochondrial volume, repaired outer membrane integrity, and promoted cristae formation. Furthermore, miR-128-3p overexpression effectively mitigated the detrimental impact of Era-induced ferroptosis on mitochondrial morphology (Fig. 4K). Furthermore, the impact of miR-128-3p on the expression of various genes, such as *SOD*, *CAT*, *NRF2*, *SLC7A11*, and *GPX4*, which are known to negatively regulate ROS and ferroptosis, was investigated. qPCR analysis revealed that the Era increased the expression of all these gene, while miR-128-3p reversed their expression (Fig. 4L, M). In contrast, miR-128-3p downregulated the expression of ferroptosis-promoting genes *ACSL4*, *PTGS2*, and *SAT1*(Fig. 4M). Additionally, a similar trend was observed in the levels of the proteins, SLC7A11 and GPX4 (Fig. 4N). These results strongly suggested that miR-128-3p weakens the sensitivity of GCs to ferroptosis.

Subsequently, the GCs were treated with miR-128-3p inhibitors (Fig. S1A). We observed a significant decrease in cell viability (Fig. S1B). Of note, miR-128-3p inhibition induced a substantial increase in intracellular ROS, Fe^2+^, and MDA levels (Fig. S1C–E), along with a decrease in GSH and MMP levels (Fig. S1F, G). Furthermore, it promoted the accumulation of lipid peroxidation (Fig. S1H). Transmission electron microscopy revealed that treatment with miR-128-3p inhibitors led to compromised mitochondrial membrane integrity, reduced mitochondrial volume, and absent cristae (Fig. S1H, I). Moreover, treatment with Fer-1, a lipid ROS scavenger known for its strong ferroptosis inhibition [38], effectively mitigated the effects of miR-128-3p inhibition. Specifically, Fer-1 treatment blocked the increase in ROS, Fe²⁺, MDA, and lipid peroxidation, and reversed the decrease in GSH and MMP levels (Fig. S1C–H). Notably, Fer-1 treatment also resulted in significant improvements in mitochondrial morphology and structural integrity in granulosa cells (Fig. S1I). Concurrently, Fer-1 significantly countered the miR-128-3p inhibitor-induced downregulation of *SOD*, *CAT*, *NRF2*, *SLC7A11*, and *GPX4*, as well as the upregulation of *ACSL4*, *PTGS2*, and *SAT1*, thereby attenuating the effects of miR-128-3p on these genes (Fig. S1J–L). In short, our results verified the miR-128-3p serves as a key regulatory factor in granulosa cell ferroptosis by modulating iron production and lipid peroxidation.

### *CSF1* is a target gene of miR-128-3p

**Fig. 5:**
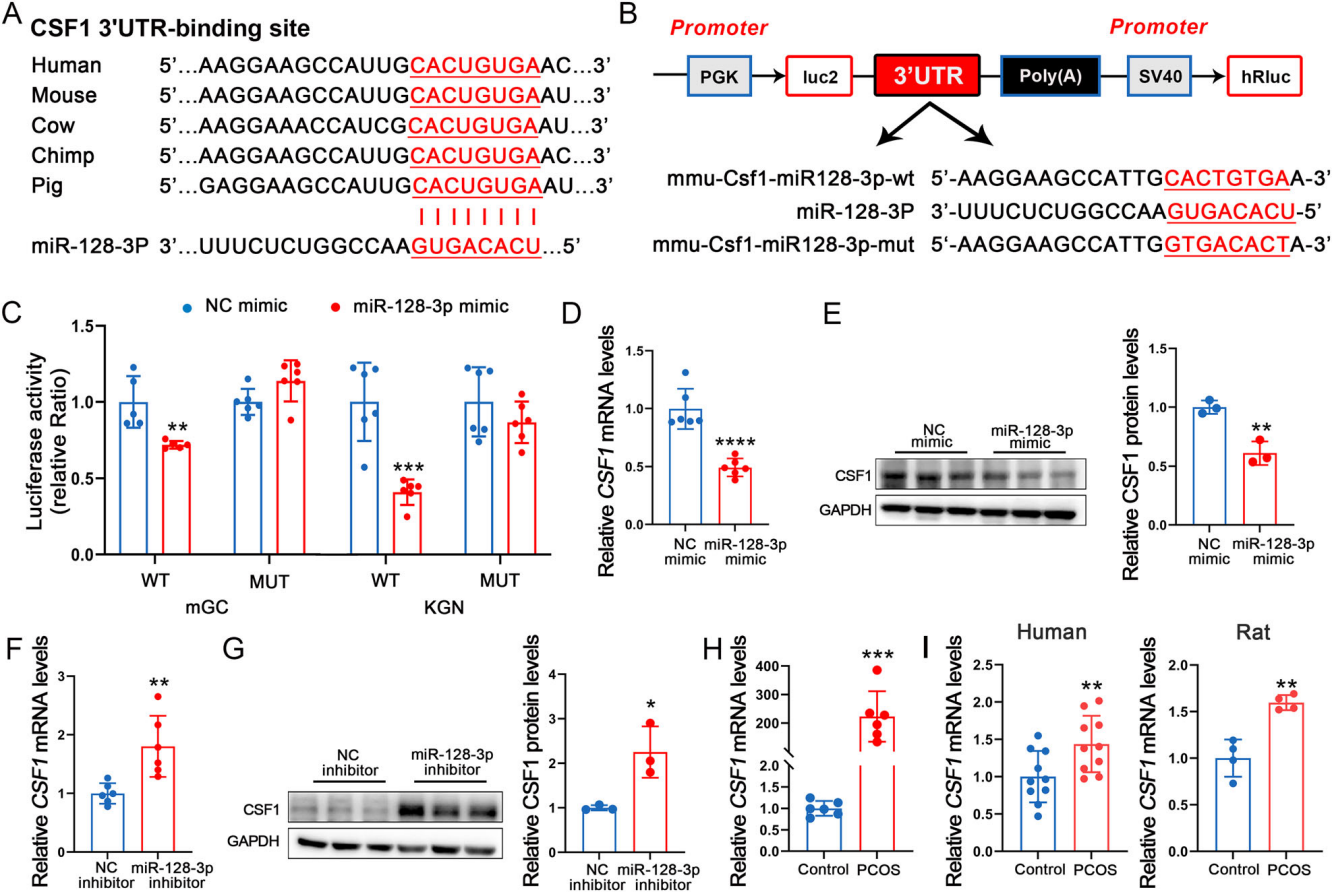
*CSF1* is a direct target gene for miR-128-3p. **A** Alignment results for the 3′ untranslated region (3′ UTR) sequence of *CSF1* from different species. **B**, **C** Dual luciferase reporter assay for miR-128-3p on the *CSF1* 3′ UTR in mGCs and KGN cells 48 h post transfection with miRNA and a luciferase reporter sequence containing mutant (MUT) *CSF1* 3′ UTR (pmiRGLO -3′ UTR of *CSF1*-MUT) or wild type (WT) *CSF1* 3′ UTR (pmiRGLO-3′ UTR of *CSF1*-WT). **D**, **E** The mRNA and protein levels of CSF1 in GCs were detected after transfection with miR-128-3p mimics. **F**, **G** The mRNA and protein levels of CSF1 in GCs were detected after transfection with miR-128-3p inhibitor. **H** The mRNA expression of CSF1 in ovary was detected by RT-qPCR. **I** The mRNA expression levels of *CSF1* in human and rat databases. Data are presented as Mean ± SD. *P < 0.05, **P < 0.01, ***P < 0.001, ****P < 0.0001.

To further elucidate the molecular mechanisms underlying the biological role of miR-128-3p in PCOS, we used the bioinformatics tools, TargetScan 8.0 and miRDB, to predict the assumed target of miR-128-3p. We found *CSF1*, a key factor involved in intracellular metabolism, oocyte meiosis, and follicular growth and maturation [39, 40], to be a potential therapeutic target. Of note, in both humans and mice, the putative binding sites for *CSF1* in 3′ UTRs, recognised by the miR-128-3p seed sequence, exhibit a high degree of conservation (Fig. 5A). To determine whether miR-128-3p targets *CSF1*, we inserted the *CSF1* 3′ UTR into pmirGLO plasmids (Fig. 5B). miR-128-3p was found to significantly reduce Luciferase activity in mGCs and KGN cells with the *CSF1* 3′ UTR-containing construct. In contrast, no effects were observed in cells with mutated *CSF1* (Fig. 5C).

Subsequent findings revealed that transfection with the miR-128-3p mimic induced a decrease in both *CSF1* mRNA and protein levels (Fig. 5D, E), whereas transfection with miR-128-3p inhibitors induced an increase in *CSF1* mRNA and protein expression (Fig. 5F, G). In addition, as opposed to the expression pattern of miR-128-3p, *CSF1* was highly expressed in the ovaries of mice with PCOS (Fig. 5H). Furthermore, publicly available datasets from ovarian granulosa cells and mesenchymal progenitor cells (MPCs) of PCOS patients (GSE138518, GSE267287) and ovarian tissues of letrozole-induced PCOS rats (GSE194398) demonstrate a significant upregulation of *CSF1* expression (Fig. 5I). In summary, these results suggest that the high expression of *CSF1* in the ovary may be a key factor in ferroptosis of ovarian GC in PCOS.

### *CSF1* mediates the effects of miR-128-3p on GCs

To validate the role of *CSF1* in ferroptosis of GCs, we transfected GCs with siRNA-*CSF1* (Fig. S2A). As anticipated, CSF1 inhibition significantly improved cell viability (Fig. S2B). This was associated with reductions in ROS, Fe²⁺, MDA, and lipid peroxidation levels, alongside increases in GSH and MMP levels (Fig. S2C–H). Furthermore, CSF1 inhibition increased mitochondrial volume (Fig. S2I) and modulated the expression of oxidative stress- and ferroptosis-related genes. Specifically, it upregulated *SOD*, *CAT*, *NRF2*, *SLC7A11*, and *GPX4* while downregulating *ACSL4*, *PTGS2*, and *SAT1* (Fig. S2J–L). Protein expression levels of SLC7A11 and GPX4 were similarly elevated following CSF1 inhibition (Fig. S2L). Notably, CSF1 inhibition also mitigated Era-induced ferroptosis (Fig. S2C–L), Thus, alterations in *CSF1* expression may be the underlying cause of ferroptosis in GCs.

**Fig. 6:**
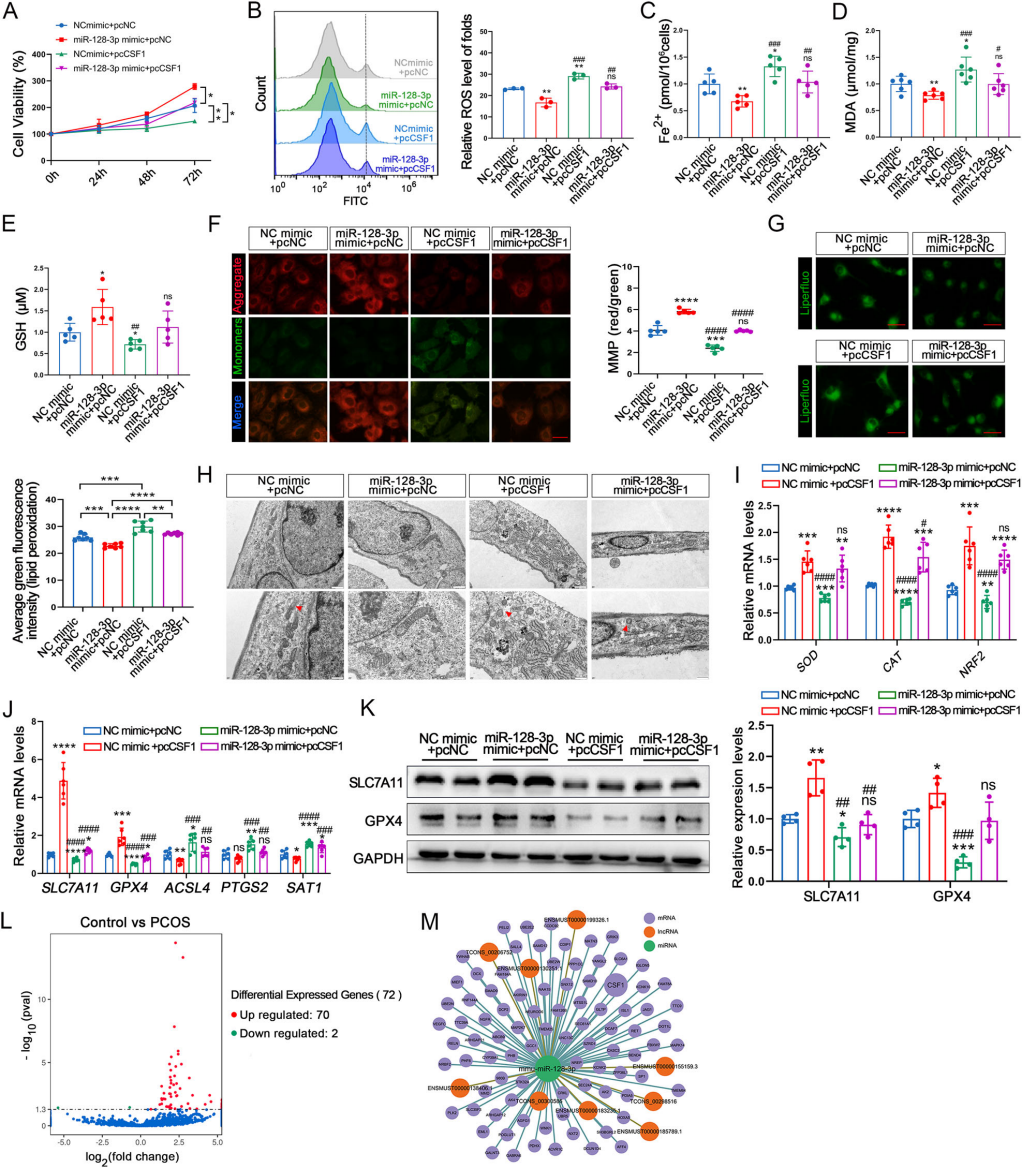
*CSF1* mediates the effects of miR-128-3p on GC ferroptosis. **A** The CCK-8 assay was performed to determine the cell viability of GC. **B** ROS levels were measured using flow cytometry. **C** Fe^2+^ content. **D** MDA levels. **E** GSH levels. **F** Determination of MMP levels in GCs through JC-1 staining. Scale bar: 30 μm. **G** Lipid Peroxidation Levels Assessed by Liperfluo Staining. Scale bar: 30 μm. **H** Low- and high-magnification images obtained by TEM, The red arrows indicate the outer mitochondrial membrane was ruptured and the mitochondrial cristae decreased or disappeared. Scale bar: 1 μm and 500 nm. **I** Relative *SOD*, *CAT*, and *NRF2* mRNA levels. **J**, **K** Ferroptosis-related gene mRNA and protein levels in GCs as determined through RT-qPCR and western blotting. **L** Volcano plot analysis showing that there are 2 downregulated and 70 upregulated lncRNAs in EXO. **M** The lncRNA-miRNA-mRNA (miR-128-3p) regulatory network. Data are presented as Mean ± SD. Ns *P* > 0.05, *P<0.05, **P < 0.01, ***P < 0.001, ****P < 0.0001 vs (NC mimic + pcNC). # *P* < 0.05, ## *P* < 0.01, ### *P* < 0.001, #### *P* < 0.0001 vs (miR-128-3p mimic + pcNC).

To investigate whether miR-128-3p-mediated regulation of ferroptosis in GCs relies on *CSF1*, we co-transfected the miR-128-3p mimic and a *CSF1* recombinant plasmid. The results revealed that GCs with overexpressed *CSF1* displayed reduced cell viability (Fig. 6A). Furthermore, through measurement of intracellular ROS levels, Fe^2+^ levels, MDA levels, GSH levels, MMP levels, lipid peroxidation levels, mitochondrial morphology, and analysis of oxidative stress- and ferroptosis-related genes, the findings suggest that the overexpression of *CSF1* weakens the inhibitory effects of miR-128-3p on lipid peroxidation and ferroptosis (Fig. 6B–K). Overall, these results confirm that *CSF1* serves as a functional target for miR-128-3p, mediating its effects on oxidative stress, and ferroptosis in GCs. Additionally, we analyzed differentially expressed lncRNAs in EXO, and the results indicated that 72 lncRNAs were differentially expressed between the two groups (Fig. 6L). Subsequently, we constructed a hypothetical lncRNA-miRNA-mRNA regulatory network based on miR-128-3p, identifying 9 lncRNAs and 88 mRNAs, including *CSF1*, that interact with miR-128-3p (Fig. 6M). Many cytoplasmic lncRNAs have been reported to act as competitive endogenous RNAs (ceRNAs) by binding to common miRNAs [41]. Therefore, we preliminarily propose that functional lncRNAs targeting the miR-128-3p/*CSF1* regulatory axis may be present in serum-derived EXOs in PCOS.

### Inhibition of miR-128-3p suppresses SLC7A11 transcription by activating the P38/JNK pathway

**Fig. 7:**
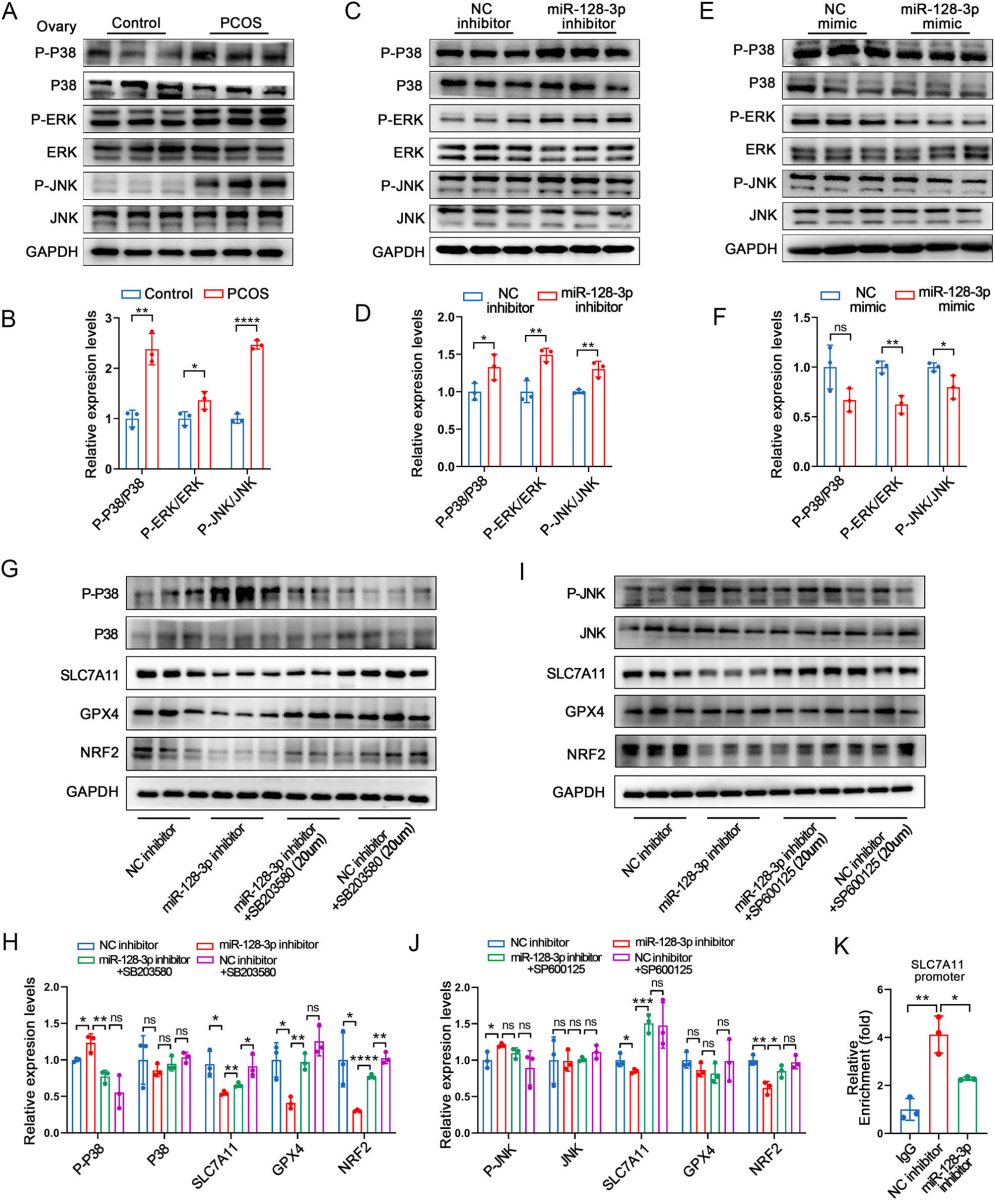
Inhibition of miR-128-3p suppresses SLC7A11 transcription through the activation of the P38/JNK pathway. **A**, **B** p38 MAPK/JNK/ERK phosphorylation levels in mouse ovaries. **C**–**F** Western blot analysis of p38 MAPK/JNK/ERK phosphorylation levels in GCs transfected with the miR-128-3p inhibitor or mimic. **G**, **H** Phosphorylated-p38, p38, SLC7A11, GPX4, and NRF2 protein levels in GCs treated with the miR-128-3p inhibitor and/or SB203580 (p38 inhibitor, 20 μM). **I**, **J** Phosphorylated-JNK, JNK, SLC7A11, GPX4, and NRF2 protein levels in GCs treated with the miR-128-3p inhibitor and/or SP600125 (JNK inhibitor, 20 μM). **K** ChIP-qPCR analysis showing NRF2 binding to the *SLC7A11* promoter. Data are presented as Mean ± SD. Ns *P* > 0.05, *P < 0.05, **P < 0.01, ****P < 0.0001.

The MAPK signalling pathway plays a key role in ferroptosis induction [30, 42, 43]. As shown in Figs. 3D and 4A, the KEGGs of all differentially expressed miRNA target genes and miR-128-3p target genes in the EXOs were enriched in the MAPK signalling pathway. This suggests that miR-128-3p downregulation may induce ferroptosis in GCs by promoting the activation of MAPK signalling. As expected, p38, JNK, and ERK phosphorylation significantly increased in the ovaries of PCOS model mice (Fig. 7A, B). To clarify whether miR-128-3p elicits its effects through the MAPK pathway, we induced miR-128-3p overexpression and inhibition in GCs cultured in vitro and analysed their effects on p38, JNK, and ERK expression. Consistent with our in vivo findings, miR-128-3p inhibition upregulated p38, JNK, and ERK phosphorylation levels (Fig. 7C, D), while its overexpression inhibited their phosphorylation levels (Fig. 7E, F).

To determine the correlation between miR-128-3p, the MAPK signalling pathway, and iron dysregulation, we treated GCs with an miR-128-3p inhibitor in combination with a p38 inhibitor (SB203580, 20 μM), a JNK inhibitor (SP600125, 20 μM), or an ERK inhibitor (PD98059, 20 μM). We found that SB203580 and SP600125 counteracted the suppressive effects of the miR-128-3p inhibitor on SLC7A11 and GPX4 expression (Fig. 7G–J). However, ERK inhibition did not reverse these effects (Fig. S3A, B). Given that *SLC7A11* expression is modulated by the transcription factor NRF2 [44], and based on our finding that NRF2 expression changes following treatment with the miR-128-3p mimic or inhibitor (Figs. 4L and S1J), we hypothesised that miR-128-3p could upregulate *SLC7A11* expression by activating NRF2. We found that the decrease in NRF2 expression following treatment with the miR-128-3p inhibitor was reversed following treatment with the p38 and JNK inhibitors (Fig. 7G–J), but not with the ERK inhibitor (Fig. S4A, B). In addition, our ChIP-qPCR analysis showed that the miR-128-3p inhibitor significantly inhibited NRF2 binding to the *SLC7A11* promoter (Fig. 7K). Thus, these results indicated that a downregulation in serum Exo-miR-128-3p expression in PCOS mice suppresses NRF2-mediated transcriptional regulation of *SLC7A11* by activating the P38/JNK pathway, thereby inducing ferroptosis in GCs.

### Overexpression of miR-128-3p can restore letrozole-induced PCOS-like features in mice

**Fig. 8:**
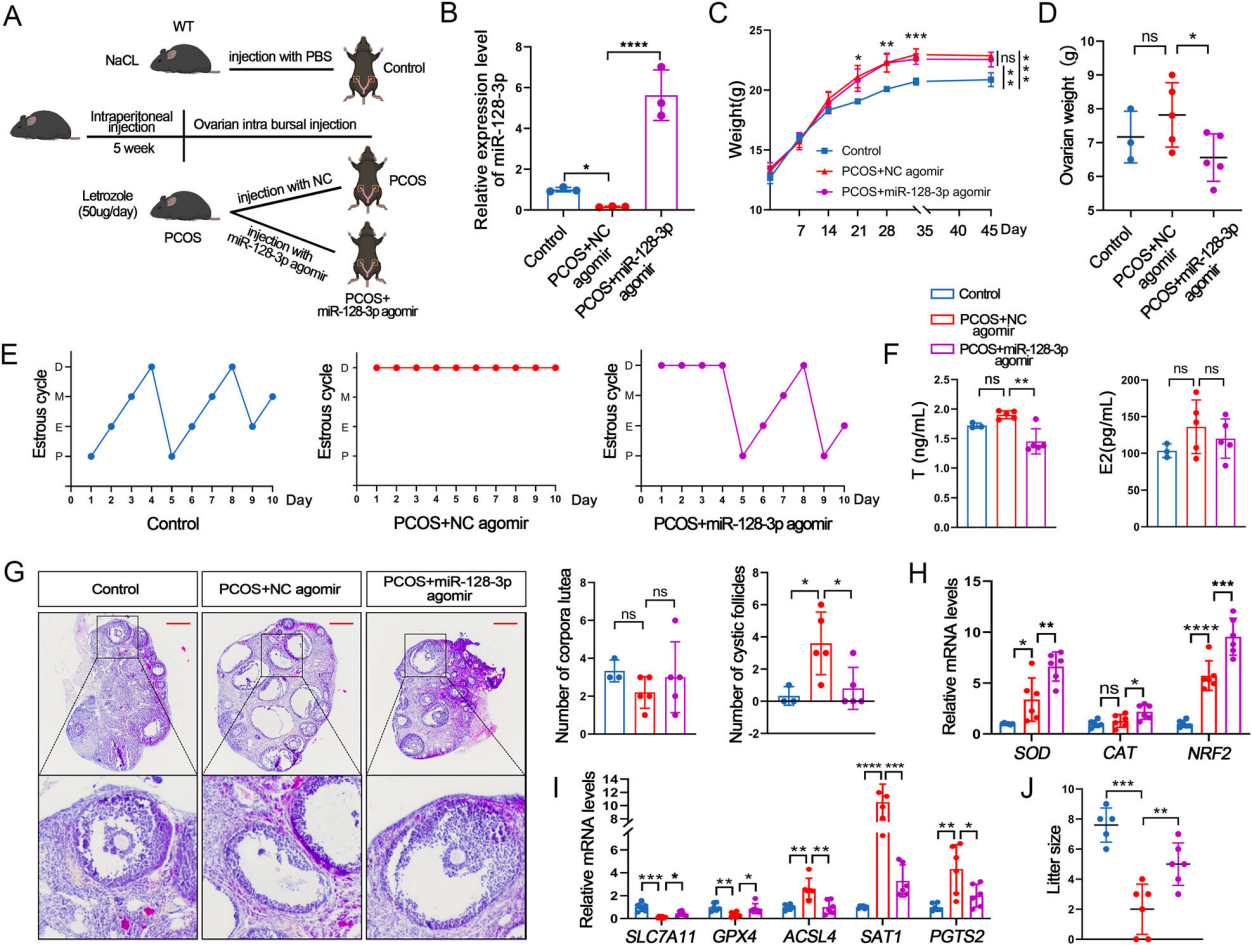
Overexpression of miR-128-3p can restore PCOS-like features in mice. **A** Schematic representation of the animal experimentation process in mice. **B** miR-128-3p expression as determined through qPCR following intrabursal injection of miR-128-3p AgomiR into mouse ovaries. **C**, **D** Body and ovary weights of mice in each group. **E** Representative estrous cycles after ovary-subcutaneous injection of miR-128-3p agomir. **F** Steroid sex hormone concentrations in mouse serum. **G** Corpus luteum and antral follicle counts following H&E staining. Scale bar: 300 μm. **H**, **I** oxidative stress- and ferroptosis-related gene mRNA expression. **J** Mouse litter size following intrabursal injection of miR-128-3p AgomiR into mouse ovaries. Data are presented as Mean ± SD. Ns *P* > 0.05, *P < 0.05, **P < 0.01, ***P < 0.001, ****P < 0.0001.

Our findings strongly indicate that miR-128-3p could serve as a novel interventional target to inhibit PCOS progression. To elucidate the biological function of this target in PCOS in vivo, as depicted in Fig. 8A, following a combination of the method specified by the manufacturer and that described by Liu et al. [45], we injected 1000 pmol miR-128-3p AgomiR into the ovaries of letrozole-induced PCOS mice. After 10 days, we measured the efficiency of miR-128-3p AgomiR overexpression in the ovaries. As expected, miR-128-3p AgomiR was highly expressed in the ovaries and did not significantly affect mouse body weight (Fig. 8B, C); however, miR-128-3p overexpression reduced ovarian weight and restored the disrupted oestrous cycle in PCOS model mice (Fig. 8D, E). As concerns serum sex hormone level detection, injection of miR-128-3p AgomiR inhibited the increase and decrease in T and E2 levels, respectively, in PCOS model mice (Fig. 8F). Furthermore, compared to NC AgomiR treatment, miR-128-3p AgomiR improved letrozole-induced cystic follicle formation but had no effect on corpora lutea counts (Fig. 8G). Moreover, compared to the control group, the expression of *ACSL4*, *PTGS2*, and *SAT1* increased, whereas those of *SOD*, *CAT*, *NRF2*, *SLC7A11* and *GPX4* decreased in the PCOS + NC AgomiR group (Fig. 8H, I). However, the expression of these genes was restored to baseline levels in the PCOS + miR-128-3p AgomiR group (Fig. 8H, I). Of note, we observed that miR-128-3p AgomiR improved the reproductive capacity of PCOS mice, as evidenced by the increase in the number of offspring produced by mice subjected to this treatment (Fig. 8J). In summary, our analyses clearly showed that miR-128-3p AgomiR attenuates letrozole-induced PCOS-like features in mice.

## Discussion

Our study demonstrated that treatment of adolescent mice with PCOS mouse serum-derived EXOs induces increased lipid peroxidation-triggered ferroptosis and the emergence of PCOS-like features in mouse ovaries. Specifically, miR-128-3p levels were lower in serum-derived exosomes from PCOS mice. The miR-128-3p mimic attenuated the Era-induced increase in intracellular ferrous iron and lipid peroxidation levels, concurrently downregulating SLC7A11 and GPX4 expression and depleting GSH reserves. Mechanistically, miR-128-3p elicited its effects by targeting *CSF1* because its overexpression inhibited the anti-ferroptosis effects of the miR-128-3p mimic. Notably, MAPK signalling was activated in PCOS mouse ovaries and in GCs treated with the miR-128-3p inhibitor. However, we found that the specific inhibitors of p38, and JNK phosphorylation, SB203580 and SP600125, attenuated the downregulatory effects of the miR-128-3p inhibitor on SLC7A11 expression. These results indicated that miR-128-3p regulated GC ferroptosis through the p38 and JNK pathways. Furthermore, we demonstrated that the p38/JNK pathway regulates *SLC7A11* transcription by inhibiting NRF2. Finally, miR-128-3p overexpression was shown to attenuate the formation of cystic follicles and improve the reproductive capacity of PCOS model mice. Collectively, our study provides preliminary evidence of the involvement of miR-128-3p packaged in serum-derived EXOs in ferroptosis in PCOS model mouse ovaries.

In this study, treatment with PCOS mouse serum-derived EXOs induced phenotypes similar to those induced following treatment with serum. These results suggested that EXOs are key factors responsible for the effects of PCOS serum on GCs. This speculation is also supported by data from previous studies which showed that EXOs are potential messenger units for ageing skeletal muscle regeneration and the improvement of Huntington’s disease in symbiotic blood exchange systems [22, 46]. Actually, under both physiological and pathological conditions, almost all cell types release EXOs containing bioactive materials. These EXOs are released into the circulatory system and are taken up by tissues where they regulate homeostasis and disease progression [22, 46]. In this study, we observed a disruption in the oestrous cycle and aberrant ovarian morphology in mice treated with PCOS mouse serum-derived EXOs. Previous studies have shown that PCOS plasma-derived EXOs affect hormonal synthesis and GC proliferation [13]. However, our study showed the occurrence of additional effects, mainly manifested as increased GC ferroptosis, in mice treated with PCOS serum-derived EXOs.

Molecular-level investigation of miRNAs within EXOs is often used for early disease detection and risk assessment. Thus, they represent a robust tool for disease prognosis determination and the formulation of innovative therapeutic strategies [47]. A literature review revealed that several miRNAs, including miR-30c, miR-146a, and miR-222, are enriched in patients with PCOS, with miR-146a showing a negative correlation with serum T levels [48]. Another study found that 34 exosomal miRNAs, including miR-18a-3p, miR-106a-5p, miR-126-3p, miR-20b-5p, and miR-146a-5p, were differentially expressed between patients with PCOS and controls [31]. Notably, a study conducted by Mohammad et al. reported no significant changes in miRNA expression in serum samples collected from individuals with PCOS [49]. Considering the variability in the findings of previous studies, interpreting miRNA expression in patients with PCOS necessitates a meticulous examination of diverse confounding factors. These differences also emphasise the need for additional exploration of miRNA profiles. In this study, we identified 35 differentially expressed miRNAs in PCOS serum-derived EXOs. Therefore, these differentially expressed miRNAs may serve as novel targets for use in diagnostic and treatment strategies. Notably, as compared to the delayed nature of imaging diagnostic strategies, peripheral blood testing provides a less invasive and more readily applicable screening strategy.

Using a gain- and loss-of-function approach, we confirmed that miR-128-3p significantly inhibits GC ferroptosis. The result study showed that miR-128-3p exerts effects against lipid peroxidation-induced ferroptosis as its overexpression decreased the levels of the lipid peroxidation product, MDA, mitochondrial damage, as well as intracellular Fe^2+^ concentrations, and inhibited ROS production, and enhanced SLC7A11 and GPX4 expression. In addition, as observed through the diverse detection approaches used in this study, including bioinformatics prediction, double-luciferase analysis, and rescue experiments, we confirmed that *CSF1*, a direct target of miR-128-3p, mediates its effects in GCs. As early as 1991, researchers explored the function of *CSF1* in GCs and confirmed its presence in the corona–cumulus complex [50]. Later Araki et al. [39]. found that the number of ovulations, antral follicles, and mature follicles were lower in osteopetrotic mutant mice (lack the coding region for the *CSF1* gene) when compared with normal litters. Furthermore, *CSF1* induces ROS production in oocytes [51], and this may explain the increase in ROS levels observed in this study following miR-128-3p inhibition and *CSF1* overexpression. Collectively, these results suggest that miR-128-3p regulates GC survival through *CSF1* targeting. LncRNAs have been reported to play an active role in the cytoplasm, where they participate in post-transcriptional regulatory processes as miRNA sponges [52]. In our study, 9 lncRNAs were identified to interact with miR-128-3p. The increased expression of *CSF1* in PCOS may be caused by the competitive binding of miR-128-3p to a certain lncRNA.

The MAPK signalling pathway plays a crucial role in ferroptosis [30, 42, 43]. Gao et al. conducted an analysis of four databases containing data from patients with PCOS and identified significant enrichment of the MAPK signalling pathway through KEGG analysis [53]. Additionally, evidence suggests that the MAPK signalling pathway is highly activated in conditions of ovarian dysfunction [54, 55]. Our KEGG enrichment analysis revealed an enrichment of the target genes of differentially expressed miRNAs in the MAPK signalling pathway. Therefore, we hypothesised that MAPK may serve as the ultimate effector for miR-128-3p/*CSF1* in the regulation of ferroptosis and GC dysfunction. MAPK constitutes a conserved group of serine-threonine kinases, primarily including the ERK, p38, and JNK subfamilies. In this study, a significant increase in ERK, p38, and JNK phosphorylation was observed in PCOS model mice and GCs treated with the miR-128-3p inhibitor. However, when GCs were treated with their respective specific inhibitors, SB203580 and SP600125 effectively reversed miR-128-3p inhibitor-induced ferroptosis. Consistent with this finding, previous studies have shown that p38 and JNK inhibition also suppresses ferroptosis in patients with leukaemia and endometriosis [56, 57]. Furthermore, the miR-128-3p inhibitor also significantly downregulated the expression levels of the nuclear factor, NRF2, and these expression levels were restored upon treatment with SB203580 or SP600125. Previous studies have shown that NRF2 mediates *SLC7A11* transcription [44]. In this study, miR-128-3p inhibition also reduced NRF2 occupancy at the *SLC7A11* promoter site. Therefore, we propose that miR-128-3p regulates the transcription of *SLC7A11* mediated by NRF2 through P38/JNK pathway, thereby influencing GC ferroptosis.

Based on these findings, we proposed a potential molecular mechanism for ferroptosis in the ovaries of PCOS model mice. PCOS mouse serum and EXOs contained in the serum are involved in survival regulation in ovarian GCs, with a significant downregulation in miR-128-3p expression. miR-128-3p deficiency increased the expression of its target gene, *CSF1*, subsequently activating the p38/JNK signalling pathway. This over-activation of p38/JNK hampers NRF2-mediated transcriptional activity of *SLC7A11*, ultimately culminating in ROS accumulation and lipid peroxidation-induced ferroptosis. Finally, we demonstrated that miR-128-3p AgomiR injection improves ovarian follicle development and mouse reproductive ability. These novel findings significantly improve our understanding of the pathogenesis of PCOS, offering promising new avenues for the identification of biomarkers and therapeutic targets.

## Materials and methods

### Animals

Three-week-old C57BL/6N mice with an average weight of 13 ± 2 g were procured from the Beijing Charles River Laboratory Animal Technology Co., Ltd (Beijing, China). Five mice were housed per cage in a facility certified for laboratory animal welfare under natural day/night lighting and constant environmental conditions. Ambient temperature and humidity were maintained at 22 ± 2 °C and 60 ± 5%, respectively. The mice were allowed unrestricted access to food and water. All animal procedures were approved by the Experimental Animal Ethics Committee of Yanbian University, China (approval number syxk2020-0009).

### Construction of the PCOS mouse model

For model establishment, we selected 21-day-old C57BL/6N female mice with an average weight of 13 ± 2 g. These mice were randomly assigned to either the PCOS or control group, with 250 mice per group. Following a week of adaptive feeding, mice in the PCOS group received 50 µg of letrozole solution (dissolved in 0.9% sodium chloride solution) per day, while mice in the control group were administered an equivalent volume of 0.9% sodium chloride solution. The weights of the mice were recorded weekly, and the mice were sacrificed after continuous treatment for five weeks. The oestrous cycles of the mice were evaluated by vaginal cytology during the last 10 days of induction. Mouse blood was collected and serum was isolated for subsequent measurement of hormone levels and isolation of EXOs. One side of the mouse ovary was fixed with 4% paraformaldehyde for haematoxylin/eosin staining and the other side was stored at −80 °C for qPCR detection. Additionally, all surviving samples were included in the analysis, and mice were randomly assigned to the experiment. However, no blinding was used for group allocation during the experiment.

### Cell culture and treatment

As previously described [45], primary mouse granulosa cells (mGCs) were isolated from 21-day-old female mice. Briefly, the mice were euthanised via cervical dislocation and their ovaries were harvested and immersed in phosphate-buffered saline (PBS, P1020, Solarbio, China). Under a microscope, the ovarian surface envelope and surrounding adipose tissue were carefully removed, washed with normal saline, and placed in serum-free DMEM/F12 (11320033, Gibco, USA). Then, the follicles were pierced with a 1-mL syringe needle to release mGCs. Subsequently, the mGCs were filtered through a 70-mesh sieve, centrifuged (1000 rpm, 8 min), and cultured in DMEM/F12 (11320033, Gibco) supplemented with 10% foetal bovine serum (FBS, A5670701, Gibco). Unless otherwise specified, all experiments were conducted using primary mGCs. KGN human ovarian granulosa cells procured from Procell Life Technology Co., LTD (Wuhan, China). The KGN cells were authenticated through short tandem repeat (STR) profiling prior to purchase and were recently confirmed to be free of mycoplasma contamination. KGN cells and mGCs were grown in DMEM/F12 (11320033, Gibco) medium supplemented with 10% FBS and 100 mg/mL penicillin-streptomycin (15140122, Gibco). Then, the cells were incubated at 37 °C in a 5% CO_2_-containing environment. Erastin (Era, HY-15763) and ferrostatin-1 (Fer-1, HY-100579) were purchased from MedChemExpress (MCE, USA).

### Isolation and identification of serum EXOs

Mouse serum-derived EXOs were isolated by differential centrifugation using a centrifuge (BECKMAN Optima XPN-100) following a previously described protocol [58]. Initially, the samples were centrifuged at 3000 × *g* for 15 min at 4 °C to eliminate cell debris. The resulting supernatant was centrifuged at 10,000 × *g* for 60 min at 4 °C to remove apoptotic bodies and microvesicles. Subsequently, the culture medium was subjected to ultracentrifugation at 100,000 × *g* for 70 min at 4 °C, and the supernatant was discarded. Then, the isolated EXOs were resuspended in PBS (P1020, Solarbio), filtered through a 0.22-μm pore filter, and centrifuged at 100,000 × *g* and 4 °C for 70 min. After discarding the supernatant, purified extracellular vesicles were precipitated and resuspended in PBS (P1020, Solarbio). The EXO concentration was determined using the bicinchoninic acid method. Next, we immediately divided the EXOs into equal parts and stored them at −80 °C in a refrigerator until use. The microstructure of serum EXOs was assessed by transmission electron microscopy (TEM, HITACHI, HT7800). The diameter of the serum EXOs was determined using a particle matrix device (NTA, ZetaView, PMX110). Serum EXO markers (CD9, CD63, and D81, as listed in Table S3) were detected by western blotting. EXOs were labelled with DiL (PKH26GL, Sigma) according to the manufacturer’s instructions. Finally, the labelled EXOs were detected by laser confocal microscopy (Olympus, OLS5100, Japan).

### Co-culture experiments

Serum and serum-derived EXOs were treated as previously described [22, 59]. Serum was incubated with GCs, with or without EXOs (20 µg/mL). To determine whether the extracted extracellular vesicles could enter GCs, they were co-cultured with DiL-labelled GCs for 24 h, photographed, and recorded using a laser confocal microscope (OLYMPUS).

### In vivo experiments on serum-derived EXOs

Three-week-old female C57BL/6N mice were adaptively fed for one week and divided into three groups, the PBS (P1020, Solarbio) injection group, the normal mouse serum EXO group (control group), and the PCOS mouse serum EXO group. Based on a previously described method [58], PBS (P1020, Solarbio) or EXOs (30 µg/mouse) were injected into the mice via the caudal vein once a week for three weeks. A vaginal smear test was performed daily from the last 10 days of treatment to observe the estrous cycle.

### Hormone assay

After 10 h of fasting, mouse blood was collected and serum was isolated. (LH, 20162400306), testosterone (T, 20162400326), follicle-stimulating hormone (FSH, 20162400317), and estradiol (E2, 20162400310) levels in serum were assayed using commercial Enzyme-Linked Immunosorbent Assay (ELISA) kits by Beijing North Institute of Biological Technology (BNIBT, Beijing, China).

### Bioinformatics analysis

Total RNA was extracted from mice in the control and PCOS groups using the TRIzol reagent (15596026CN, Invitrogen). Following the protocol recommended by the vendor, we performed 50-bp single-end sequencing on an Illumina NovaSeq 6000 device (Beijing Allwegene Technology Co., Ltd, Beijing, China). Differential analysis miRNAs with |log_2_ (fold change)| ≥1 and *p* < 0.05, as determined using the R package, were selected. From the Gene Expression Omnibus (GEO, https://www.ncbi.nlm.nih.gov) Download human miRNA expression profiles (GSE142819), as well as human (GSE138518) and rat (GSE194398) mRNA expression profiles.

### Vector construction and luciferase reporter assays

Construction of the *CSF1* plasmid involved synthesis of the *CSF1* coding sequence through PCR amplification and subsequent cloning into pcDNA-3.1. Subsequently, Lipofectamine 3000 (L3000015, Invitrogen) was used to transfect inhibitors, mimics, siRNAs, and *CSF1* plasmids (Table S1, Gene Pharma, Suzhou, China) into both mGCs and KGN cells. For the double-luciferase reporter assay, mimics and *CSF1* 3′ untranslated region (3′ UTR, normal or mutated) reporter plasmids were co-transfected and cloned into the pmiRGLO vector (Gene Pharma). Luciferase activity was assessed 48 h post transfection using a Microplate Reader (Thermo Fisher Scientific) and determined by comparing the Firefly/Renilla luciferase ratio.

### RNA extraction and RT-qPCR

Total RNA was extracted from tissues and cells using the TRIzol extraction reagent kit (DP424, Tiangen, China) according to the manufacturer’s instructions. The FastKing one-step method was used to remove the first strand of genomic cDNA synthesis premix (KR118, Tiangen) to produce complementary DNA. RT-qPCR reactions were performed using the Eco 48 system (PCRmax, UK) and the FastStart Universal SYBR Premix ExTaq (4913850001, Roche, Germany). The mRNA expression level was measured with *β-actin* as the internal reference gene.

For miRNA quantification, total RNA was extracted using the miRNeasy Mini Kit (169029110, Qiagen, Hilden, Germany). Subsequently, a miRcut-enhanced miRNA cDNA first-strand synthesis kit (KR211, Tiangen) was used for reverse transcription. The miRNAs were quantified using the miScript SYBR Green PCR Kit (FP411, Tiangen). miRNA expression levels were determined using U6 as an internal control. The primer sequences are shown in Table S2.

### Western blot analysis

Total proteins from ovarian tissues or KGN cells were isolated using a RIPA buffer (P0013B, Beyotime, China). Protein concentration was determined using a bicinchoninic acid kit (P0010, Beyotime). Equal amounts of total protein were separated by SDS polyacrylamide gel electrophoresis and transferred onto a PVDF membrane (IPVH00010, Merck Millipore, USA). The membrane was blocked with 5% skim milk for 1 h and incubated overnight with primary antibodies at 4 °C. On the following day, the membrane was incubated with the appropriate secondary antibodies at room temperature for 1 h and washed thrice with TBST at 5-min intervals. Chemiluminescence signals were detected using the ECL detection reagent (P0018S, Beyotime). All antibodies used in this study are listed in Table S3. The JNK (SP600125), ERK (PD98059), and p38 (SB203580) inhibitors were purchased from MCE.

### Cell viability assays

GC viability was assessed using cell counting Kit 8 (CCK-8, C0038, Beyotime). The cell suspension was inoculated in a 96-well culture plate. After culturing for 24–72 h, the medium was removed, and 100 μL of complete medium containing 10 μL of CCK8 reagent was added to the cells and cultured at 37 °C for 2 h in a CO_2_ incubator. Absorbance was measured at 450 nm and cell viability was calculated.

### Malondialdehyde, liperfluo and Fe^2+^ detection

A lipid peroxidation MDA assay kit (S0131M, Beyotime) was used to measure relative MDA content, which is a quantitative biomarker for ferroptosis, in the cell lysate according to the instructions of the manufacturer. Briefly, KGN cells were lysed using a lysis buffer (P0013B, Beyotime) and centrifuged at 12,000 × *g* for 10 min. Next, 100 μL of the supernatant was mixed with 200 μL of MDA working solution, incubated at 100 °C for 15 min, and then cooled to room temperature. Absorbance was measured at 532 nm using an enzyme-labelled instrument (Thermo Fisher Scientific).

Cells were cultured in 24-well plates and subjected to induction for 24 h under standard conditions. After induction, the culture medium was removed, and the cells were washed once with serum-free MEM medium. A 5 μmol/L Liperfluo solution, prepared in serum-free MEM medium, was added to each well at a volume of 400 μL, followed by incubation at 37 °C in a humidified atmosphere with 5% CO_2_ for 30 min. The staining solution was then removed, and the cells were washed twice with 400 μL of HBSS to eliminate residual dye. Lipid peroxidation was observed using a fluorescence microscope (Olympus, IX73, Japan), with excitation and emission wavelengths set at 488 nm and 500–550 nm, respectively.

Following the instructions of the manufacturer, ferrous iron concentrations in KGN cell lysates were assessed using an iron assay kit (ab83366, Abcam, California, UK). Subsequently, to determine ferrous iron levels, absorbance at 593 nm was measured enzyme-labelled instrument (Thermo Fisher Scientific).

### Reactive oxygen species (ROS) assay

Following the instructions of the manufacturer, 1 × 10^5^ cells were seeded per well in a 24-well plate for subsequent treatment. Transfection was performed when the cell density reached ~60%. A 5 μM DCFH-DA (4091-99-0, MCE) staining solution was prepared in pre-warmed serum-free medium. The culture medium was discarded, and 500 μL of the staining solution was added to each well. The cells were incubated at 37 °C in a 5% CO_2_ incubator for 30 min. After incubation, the cells were washed twice with PBS (P1020, Solarbio), digested with trypsin (T4049, Sigma), and centrifuged. Finally, the cells were resuspended in a serum-free medium for flow cytometric analysis (BD Accuri®C6 Plus, USA) and data were analysed using the FlowJo (TreeStar) software.

### Glutathione (GSH) detection

Forty-eight hours after transfection, GCs were subjected to a GSH/GSSG-Glo assay (V6611, Promega, USA) according to the manufacturer’s instructions, and GSH concentration was measured using a microplate reader from Thermo Fisher Scientific.

### Mitochondrial membrane potential measurement

The MMP (ΔΨm) of GCs was measured using the JC-1 assay kit (C2006, Beyotime). Following the instructions of the manufacturer, treated GCs were incubated with 10 μg/mL JC-1 staining solution at 37 °C for 20 min and then washed twice with PBS before observation under a fluorescence microscope (Olympus, IX73, Japan). JC-1 aggregates in functional mitochondria with high MMP and emits a red fluorescence. Conversely, JC-1 is released from depolarised mitochondria with low MMP in the form of monomers that emit green fluorescence. In this study, fluorescence levels were quantified using the ImageJ software and ΔΨm was determined by calculating the red fluorescence/green fluorescence ratio.

### Transmission electron microscopy

The cell samples were initially fixed in 2.5% glutaraldehyde (pH 7.4) for 2 h. After washing three times with 0.1M phosphate buffer (pH 7.2), the samples were post-fixed in 1% osmium tetroxide at 4 °C for 2 h. The cells were then dehydrated through a graded ethanol series and infiltrated with Epon-Araldite resin, followed by embedding in a mold for polymerization. After semi-thin sections were prepared for orientation, ultrathin sections were cut for microstructural analysis. The sections were counterstained with 3% uranyl acetate and 2.7% lead citrate. Finally, the ultrastructure of the cells was examined using a JEM-1400 transmission electron microscope.

### Chromatin immunoprecipitation (ChIP) assay

ChIP assays were conducted using the Chromatin immunoprecipitation (ChIP) test kit (17-295, Sigma, USA) according to the instructions of the manufacturer. DNA fragments crosslinked with NRF2 were immunoprecipitated using anti-NRF2 antibodies, with anti-mouse IgG serving as the negative control. qPCR was performed to detect DNA fragments from the *SLC7A11* promoter region. The primers used are listed in Supplementary Table S2.

### miR-128-3p AgomiR injection into the ovarian bursa

miR-128-3p AgomiR (sense: 5′-UCACAGUGAACCGGUCUCUUU-3′, antisense: 5′-AGAGACCGGUUCACUGUGAUU-3′) and NC AgomiR (sense: 5′-UUCUCCGAACGUGUCACGUTT-3′, antisense: 5′-ACGUGACAOGUUCGGAGAATT-3′) were obtained from GenePharma (Suzhou, China). Based on a combination of the method described by the supplier and that described by Liu et al. [45], the back skin and muscles of mice were incised following isoflurane anaesthesia, and both ovaries were carefully exposed. Next, ovarian sacs were injected with either miR-128-3p AgomiR or NC AgomiR (1000 pmol/ovary). Subsequently, the treated mice were housed individually and their vaginal smears were monitored continuously for 10 days from the second day following surgery. On the 10th post operative day, all mice were euthanised, and their ovaries and serum samples were collected.

### Fertility assessment

To assess fertility, female mice were paired with mature male mice at a 1:1 ratio. The following day, the vaginal suppository was checked to determine whether mating had occurred. The male mice were removed after 10 days, and the fertility of the female mice was assessed within 35 days.

### Statistical analysis

Data are expressed as mean ± SD. The experiments were repeated at least three times. All data were analysed using SPSS version 25.0 (IBM, USA) or GraphPad Prism 9.5 (GraphPad Software, USA). All data followed a normal distribution as determined by the Shapiro–Wilk test. Differences between groups were analyzed using a two-tailed Student’s *t*-test or one-way analysis of variance (ANOVA). Differences were considered significant at *P* < 0.05 (*), *P* < 0.01 (**), *P* < 0.001 (***), and *P* < 0.0001 (****). Non-significant differences, i.e. *P* > 0.05, were indicated with N.S.

## Supplementary information

Supplementary figures

Supplementary Tables

Full and uncropped western blots

## Data Availability

The raw reads of mouse miRNA-seq gene sequences have been submitted to the NCBI BioSample database (accession SUB14496639). All sample metadata and intermediate analysis files are available at (https://data.mendeley.com/datasets/5tsfvkkt94/1).
