# Supplementary material for: Decreased miR-128-3p in serum exosomes from polycystic ovary syndrome induces ferroptosis in granulosa cells via the p38/JNK/SLC7A11 axis through targeting CSF1
Source: Cell Death Discov. 2025 Feb 18;11:64. doi: 10.1038/s41420-025-02331-0 (PMC11836375; doi:10.1038/s41420-025-02331-0)
Supplement: Supplementary file 1 — Supplementary figures [file 41420_2025_2331_MOESM1_ESM.pdf]

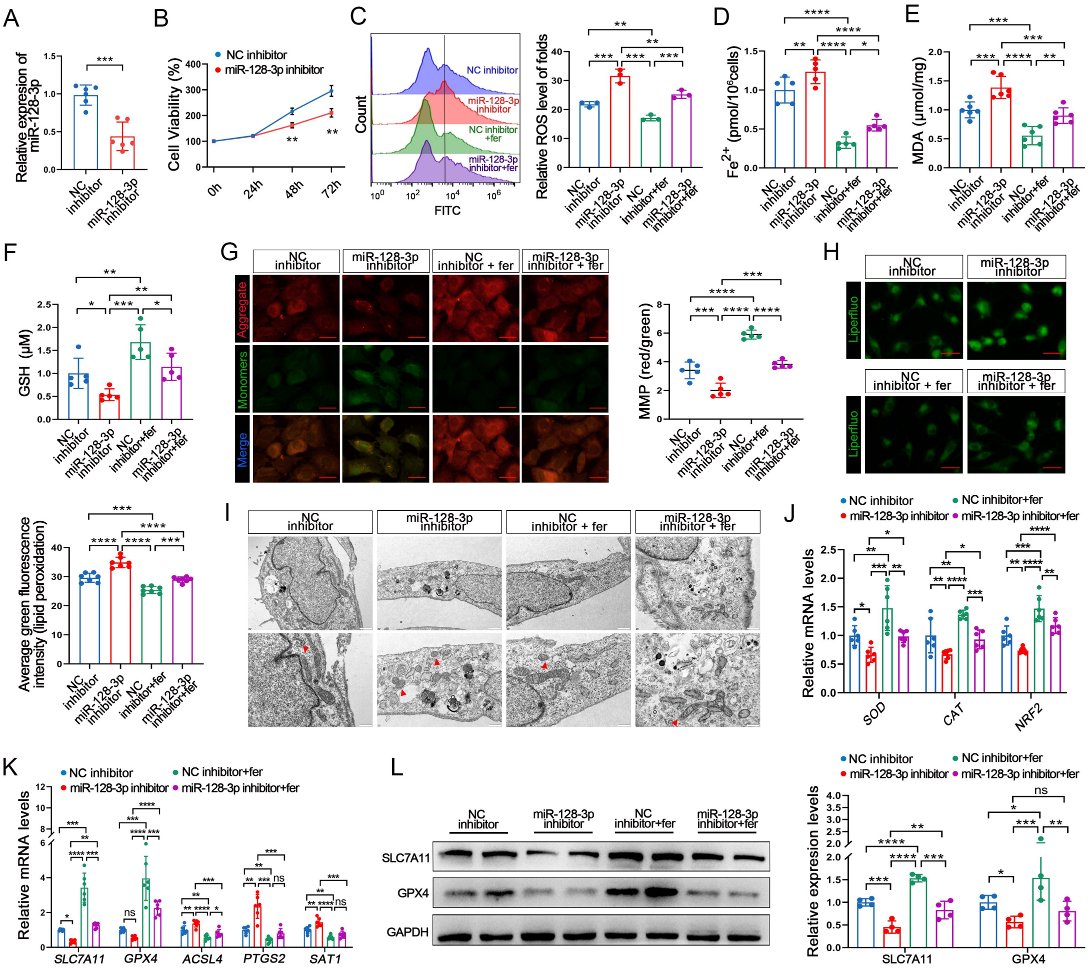

Figure S1. Inhibition of miR-128-3p promotes GC ferroptosis

(A) miR-128-3p expression levels in GCs following transfection with the miR-128-3p inhibitor. (B) GC viability following treatment with the miR-128-3p inhibitor. (C–G) Intracellular ROS, Fe^{2+}, MDA, GSH, and MMP levels in GCs treated with the miR-128-3p inhibitor and ferrostatin-1. Scale bar: 30 μm. (H) Lipid Peroxidation Levels Assessed by Liperfluo Staining. Scale bar: 30 μm. (I) Low- and high-magnification images obtained by TEM, The red arrows indicate the outer mitochondrial membrane was ruptured and the mitochondrial cristae decreased or disappeared. Scale bar: 1 μm and 500 nm. (J) Relative mRNA levels of oxidative stress-related genes. (K and L) Ferroptosis-related gene mRNA and protein levels in GCs treated with the miR-128-3p inhibitor and ferrostatin-1. Data are presented as Mean ± SD. Ns P>0.05, * P<0.05, ** P<0.01, *** P<0.001, **** P<0.0001.

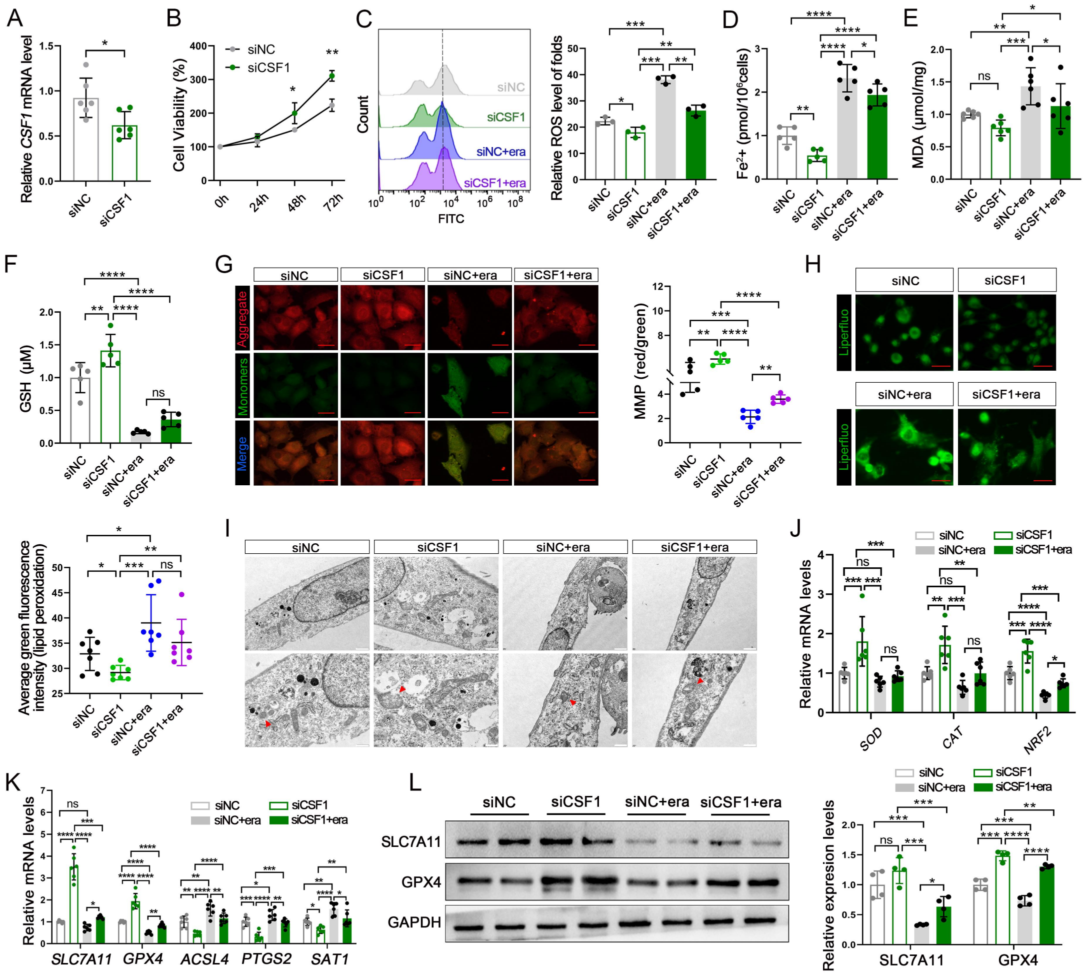

Figure S2. Inhibition of *CSF1* suppresses GC ferroptosis.

(A) Relative *CSF1* mRNA expression levels following transfection with siCSF1. (B) A CCK-8 assay was performed to determine the viability of GC. (C) ROS levels in the indicated cells were measured using flow cytometry following DCFH-D staining. (D) Fe^{2+} content (E) MDA levels. (F) GSH levels. (G) Measurement of MMP levels in GCs using JC-1 staining. Scale bar: 30 μm. (H) Lipid Peroxidation Levels Assessed by Liperfluo Staining. Scale bar: 30 μm. (I) Low- and high-magnification images obtained by TEM, The red arrows indicate the outer mitochondrial membrane was ruptured and the mitochondrial cristae decreased or disappeared. Scale bar: 1 μm and 500 nm. (J) Relative *SOD*, *CAT*, and *NRF2* mRNA levels. (K and L) Ferroptosis-related gene mRNA and protein levels as determined via RT-qPCR and western blotting, respectively. Data are presented as Mean ± SD. Ns *P*>0.05, * *P*<0.05, ** *P*<0.01, *** *P*<0.001, **** *P*<0.0001.

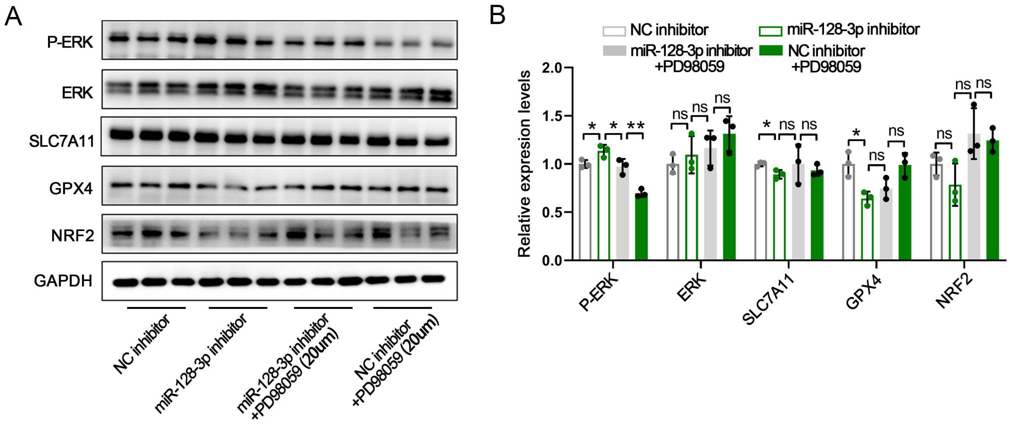

Figure S3. The effect of the ERK signaling pathway on ferroptosis in GCs

(A and B) Protein levels in GCs were measured following treatment with PD98059 (an ERK inhibitor, 20 μM) alone or in different combinations. Data are presented as Mean ± SD. Ns *P*>0.05, * *P*<0.05, ** *P*<0.01.
