# Supplementary material for: Decreased miR-128-3p in serum exosomes from polycystic ovary syndrome induces ferroptosis in granulosa cells via the p38/JNK/SLC7A11 axis through targeting CSF1
Source: Cell Death Discov. 2025 Feb 18;11:64. doi: 10.1038/s41420-025-02331-0 (PMC11836375; doi:10.1038/s41420-025-02331-0)
Supplement: Supplementary file 2 — Supplementary Tables [file 41420_2025_2331_MOESM2_ESM.pdf]

Table S1. Sequences of the oligonucleotides used in the study

Designation	Sequence 5’ to 3’
NC mimic	UUCUCCGAACGUGUCACGUTT
	ACGUGACACGUUCGGAGAATT
miR-128-3p mimic	UCACAGUGAACCGGUCUCUUU
	AGAGACCGGUUCACUGUGAUU
NC inhibitor	CAGUACUUUUGUGUAGUACAA
miR-128-3p inhibitor	AAAGAGACCGGUUCACUGUGA
NC siRNA	UUCUUCGAACGUGUCACGUTT
	ACGUGACACGUUCGGAGAATT
CSF1 siRNA	GGAGACUUCAUGCCAGAUUTT
	AAUCUGGCAUGAAGUCUCCTT

Table S2. Sequences of primers used in the study

Gene	Sequence 5’ to 3’
β-actin	Forward: GTACCACCATGTACCCAGGC
	Reverse: AACGCAGCTCAGTAACAGTC
SLC7A11	Forward: GTTCAGACGATTGTCAGACAGAA
	Reverse: GGCAGATGGCCAAGGATTTG
GPX4	Forward: CCTGGCTGGCACCATGT
	Reverse: CACACGCAACCCCTGTACTT
CSF1	Forward: CTCTAGCCGAGGCCATGTGGA
	Reverse: GACTAGGATGATGCCCGGCAC
ACSL4	Forward: CCACACTTATGGCCGCTGTT
	Reverse: GACTAGGATGATGCCCGGCAC
PTGS2	Forward: CTGCGCCTTTTCAAGGATGG
	Reverse: GGGGATACACCTCTCCACCA
SAT1	Forward: TGACCCATGGATTGGCAAGT
	Reverse: CAGCGACACTTCATGGCAAC
SOD	Forward: TGTCCATTGAAGATCGTGTGAT
	Reverse: TCATCTTGTTTCTCATGGACCA
CAT	Forward: CACCTTCAAGTTGGTTAATGCA
	Reverse: CATGACCTGGATGTTAAAACGTC
NRF2	Forward: TGGGCTGTGTGTTCTGAGTATG
	Reverse: TTGCTGCGCAACTGATCAAC
SLC7A11(ChIP)	Forward: GGGCGTCATAGCCTTTCTTG
	Reverse: TGAGCAACAAGCTCCTCCTG
let-7f-5p	Forward: CGCGCCGCTGAGGTAGTAGATTGTATAGTT
miR-1b-5p	Forward: CGCCGCCGTACATACTTCTTTACATTCCA
miR-128-3p	Forward: CGCCGCCGTACATACTTCTTTACATTCCA
miR-486b-3p	Forward: CCGGGGCAGCTCAGTACAGGA
RNU6B	Forward: CGCGCAAGGATGACACGCAAATTCG

Table S3. Antibodies used in the study

Antibody	Manufacturer	CAT	Dilution ratio
CD 9	Proteintech	20597-1-AP	1:2000
CD 63	Proteintech	25682-1-AP	1:2000
CD 81	Bioss	bs-6934R	1:2000
GAPDH	Proteintech	60004-1-Ig	1:2000
GPX4	Proteintech	67763-1-Ig	1:1000
SLC7A11	Proteintech	26864-1-AP	1:1000
NRF2	Proteintech	16396-1-AP	1:2000
ERK1/2	Cell Signaling	9101S	1:2000
P-ERK1/2	Cell Signaling	9102S	1:2000
JNK	Cell Signaling	9251	1:2000
P-JNK	Cell Signaling	9252	1:2000
P38 MAPK	Bioss	bs-28027R	1:2000
P-P38 MAPK	Bioss	bs-5476R	1:2000
Anti-Mouse IgG(H+L)	Beyotime	A0216	1:2000
Anti-Rabbit IgG(H+L)	Beyotime	A0208	1:2000
