# Supplementary material for: Decreased miR-128-3p in serum exosomes from polycystic ovary syndrome induces ferroptosis in granulosa cells via the p38/JNK/SLC7A11 axis through targeting CSF1
Source: Cell Death Discov. 2025 Feb 18;11:64. doi: 10.1038/s41420-025-02331-0 (PMC11836375; doi:10.1038/s41420-025-02331-0)

Figure 1F

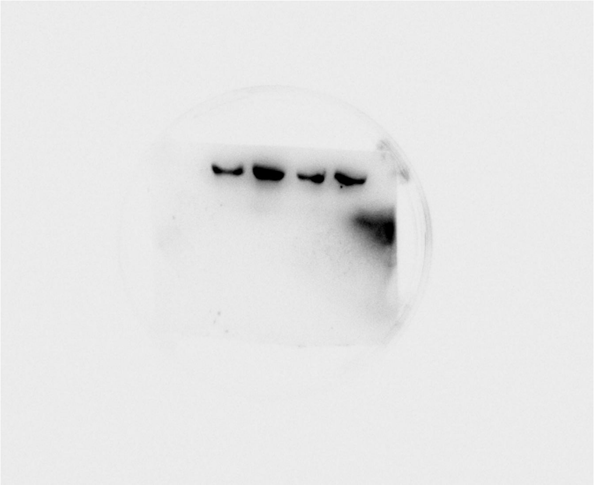

CD9

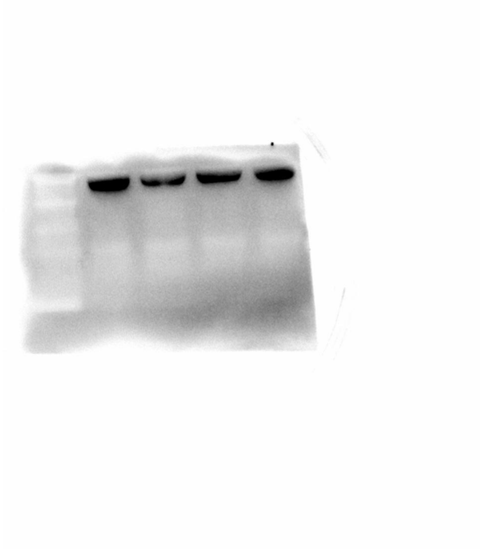

CD63

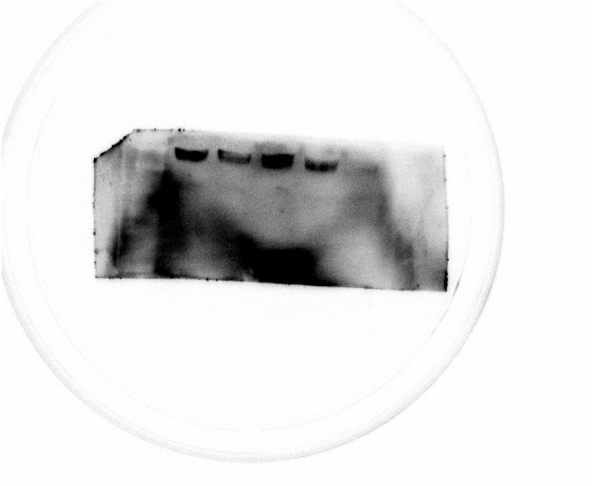

CD81

Figure 4N

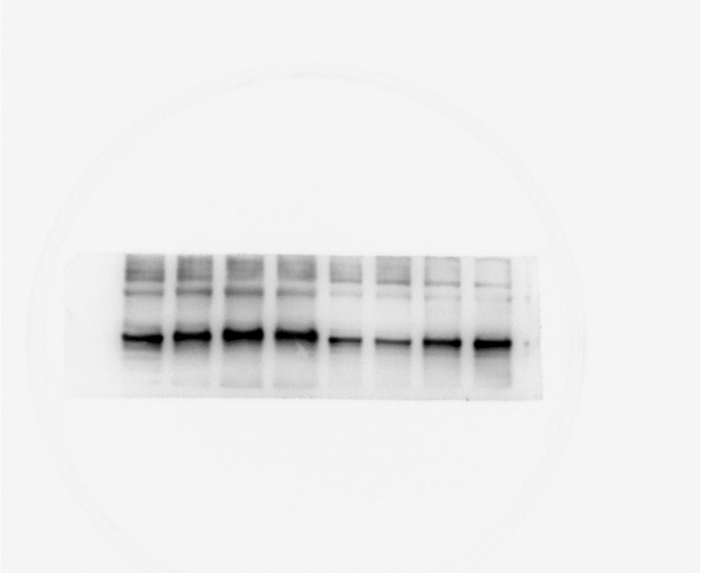

SLC7A11

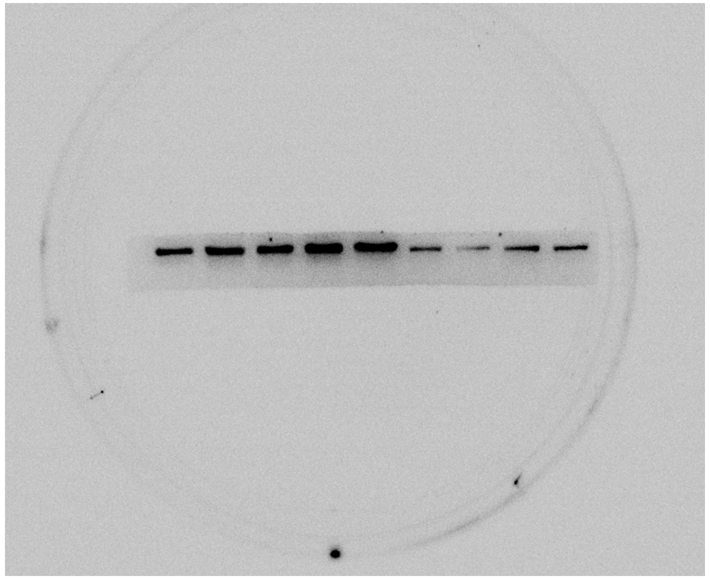

GPX4

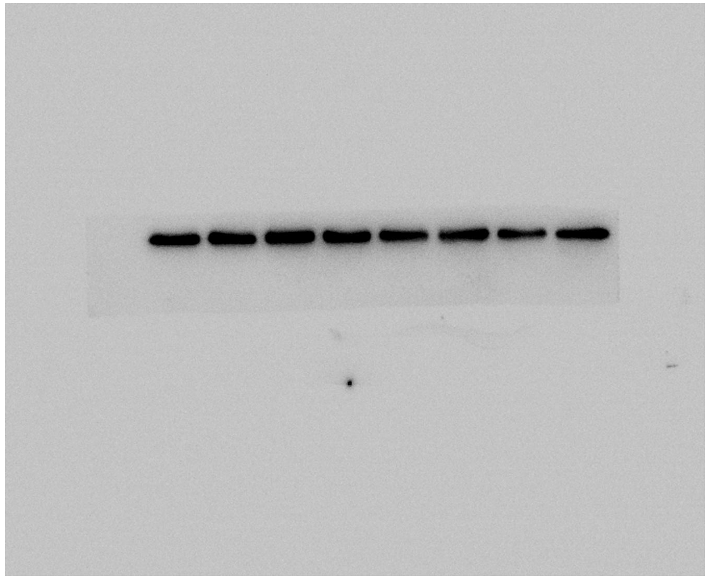

GAPDH

Figure 5E

CSF1

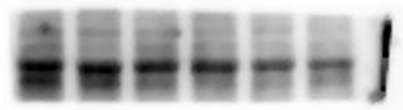

GAPDH

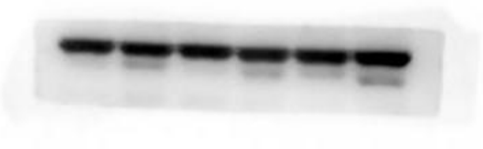

Figure 5G

CSF1

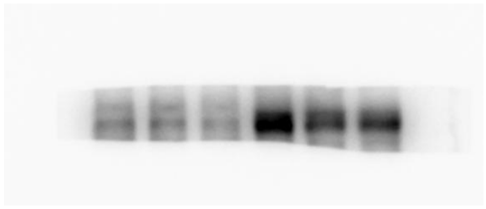

GAPDH

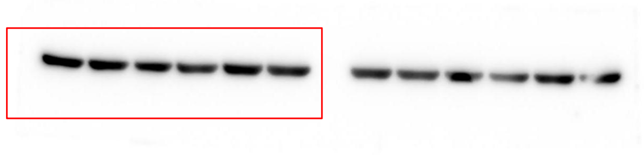

Figure 6K

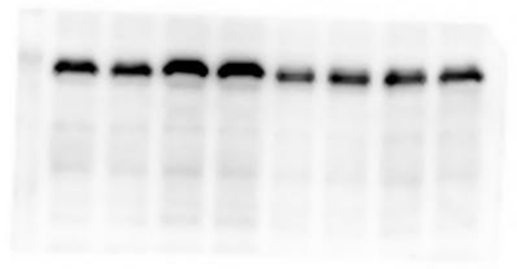

SLC7A11

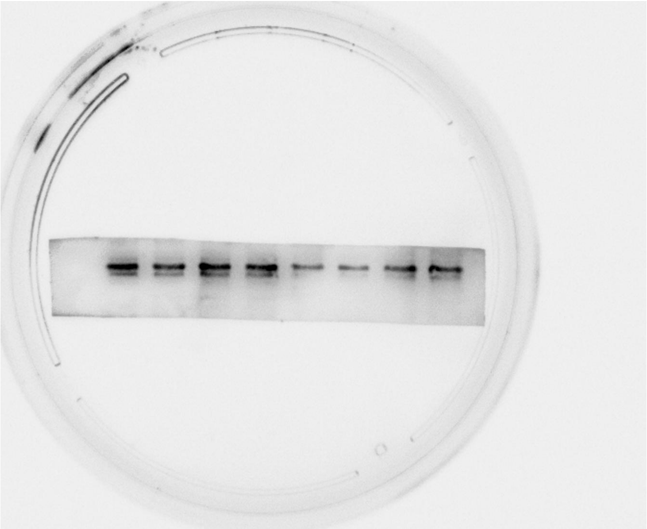

GPX4

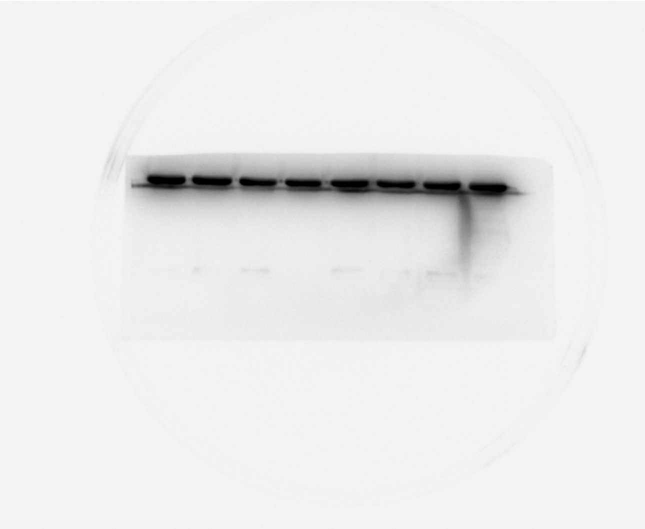

GAPDH

Figure 7A

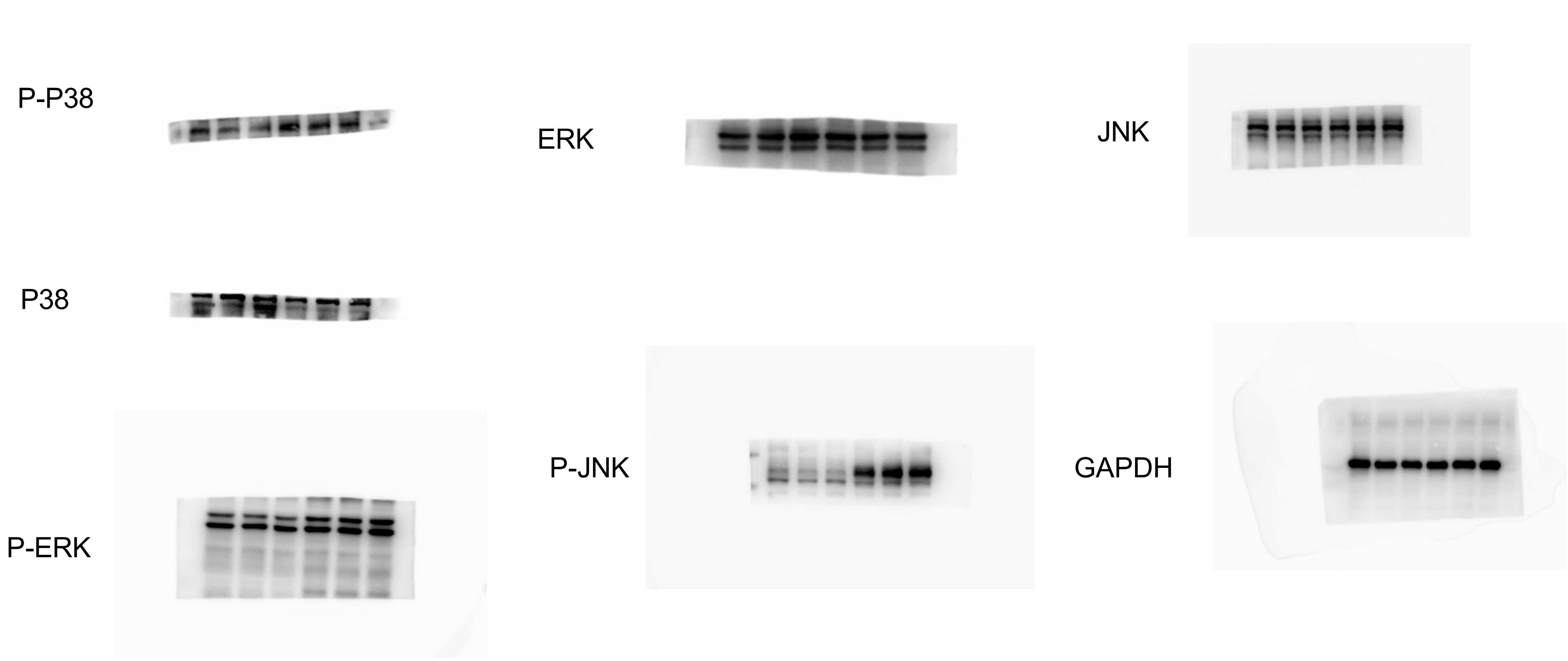

Figure 7C

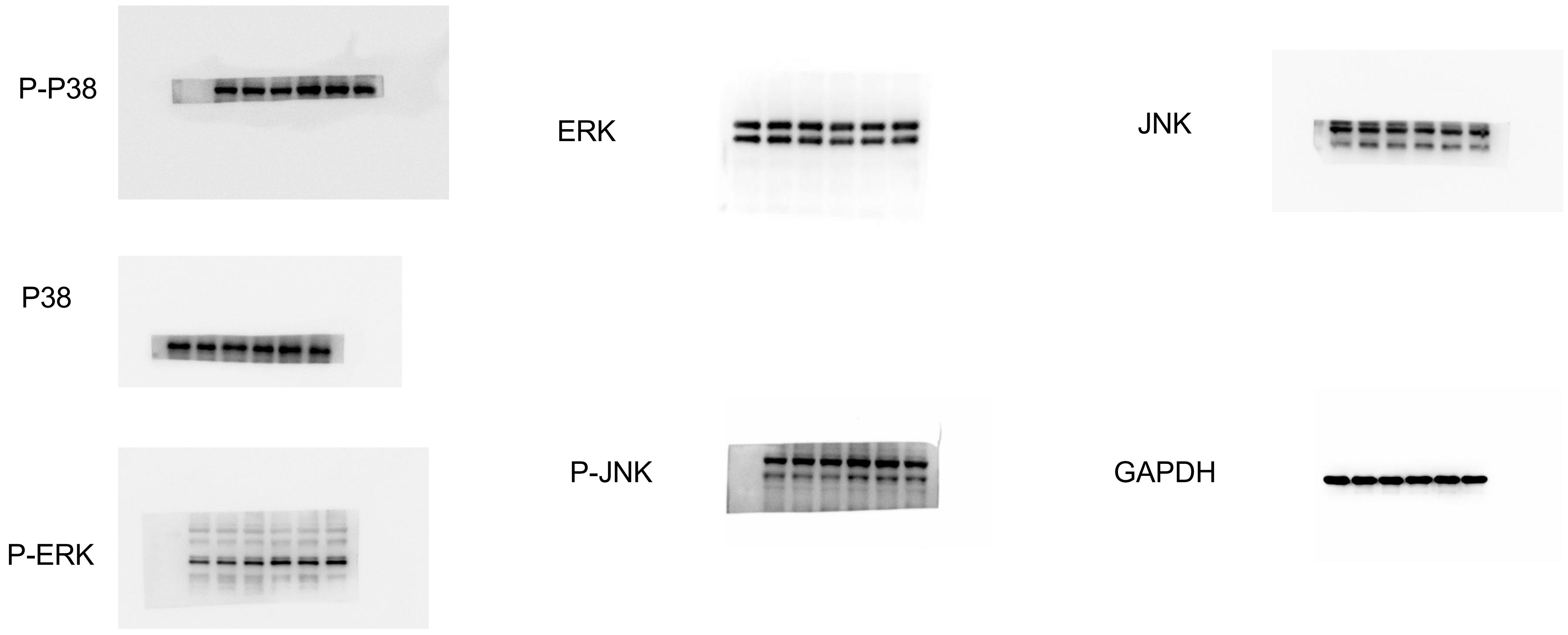

Figure 7E

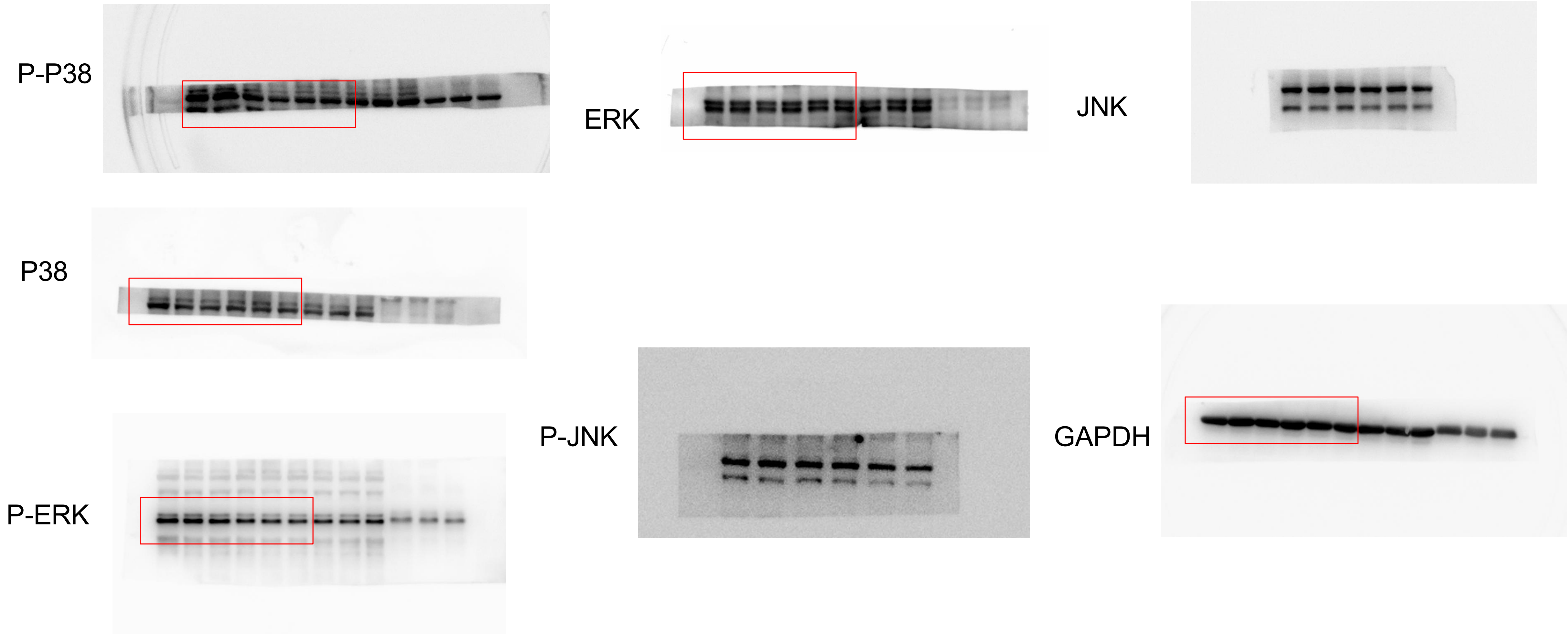

Figure 7G

P-P38

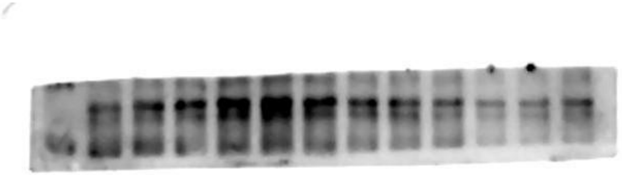

P38

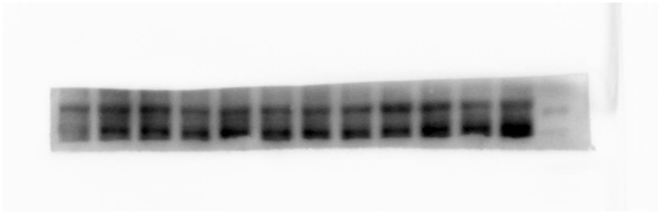

SLC7A11

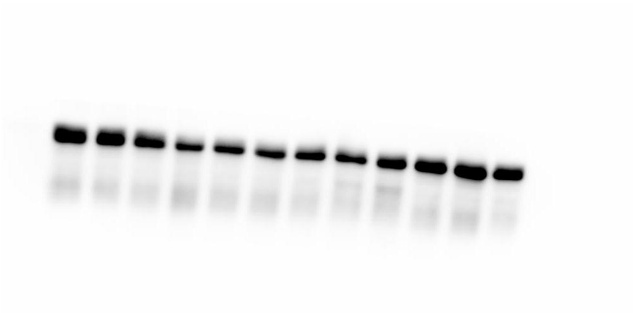

GPX4

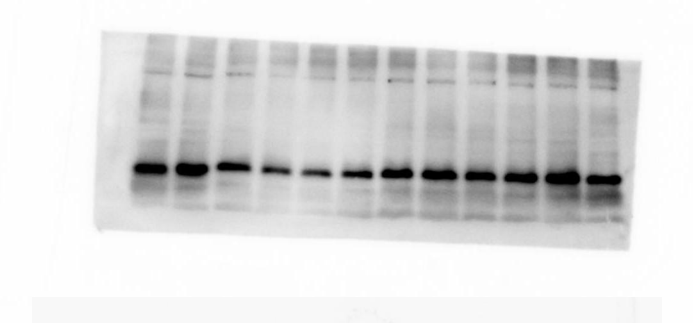

NRF2

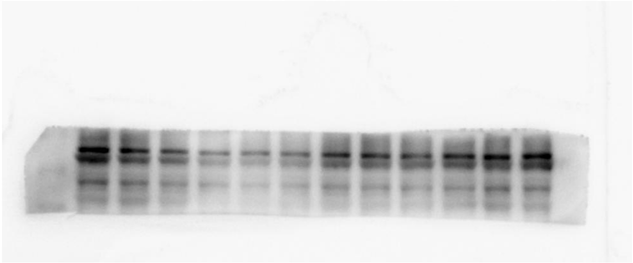

GAPDH

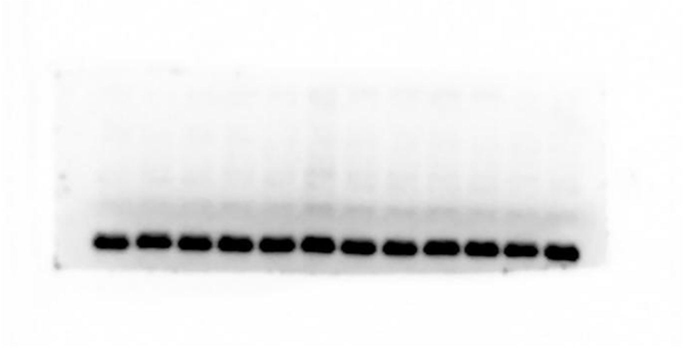

Figure 7I

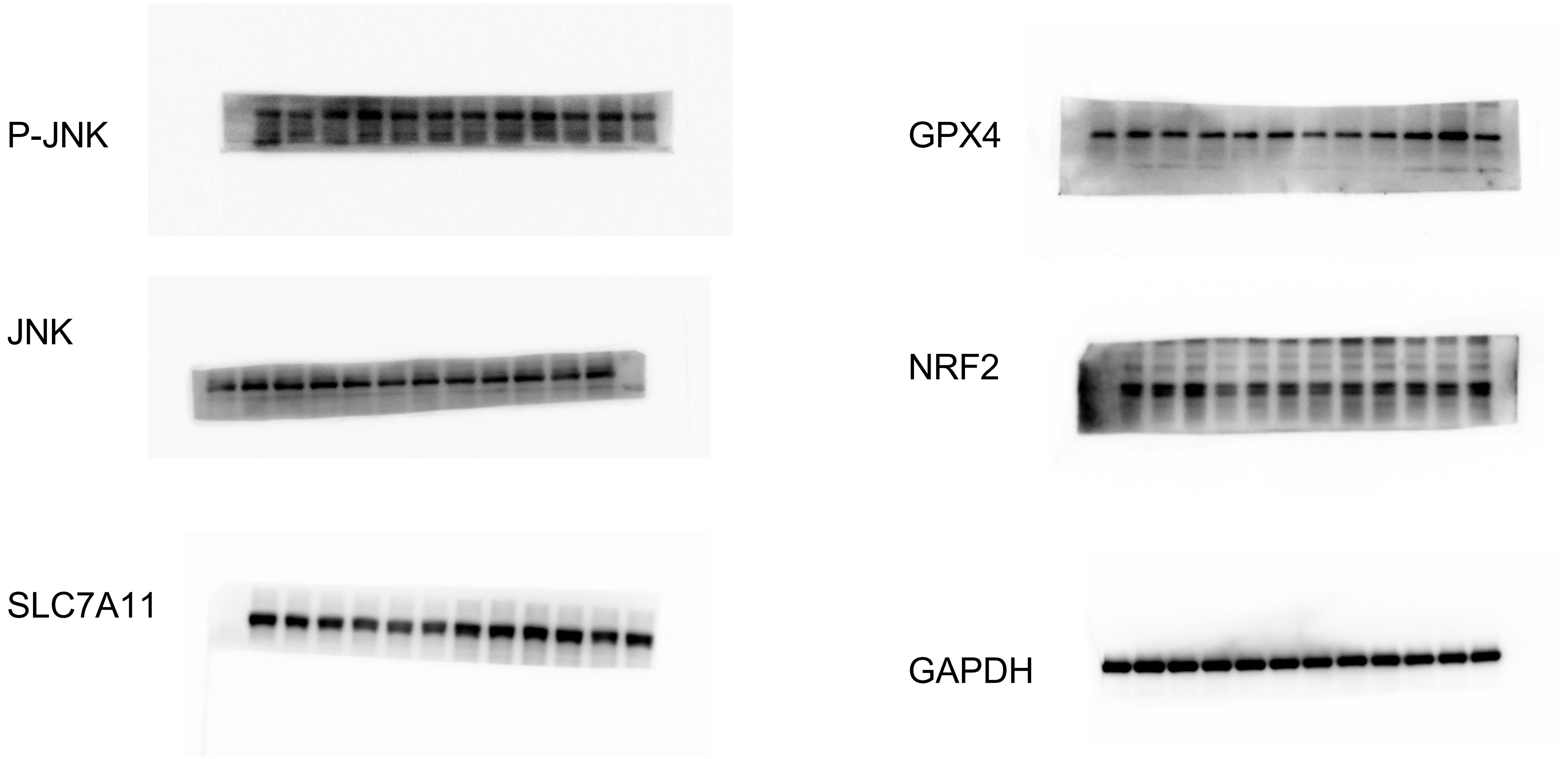

Figure S1L

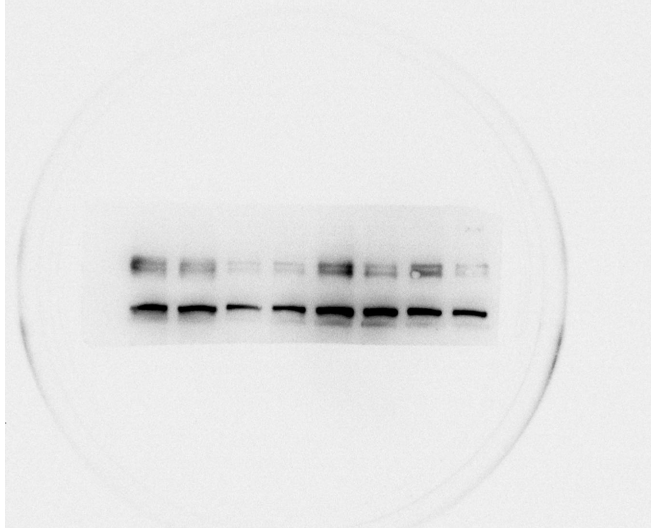

SLC7A11

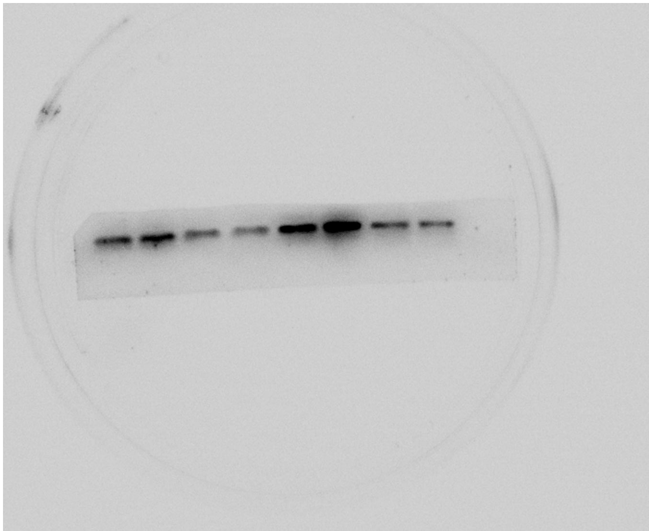

GPX4

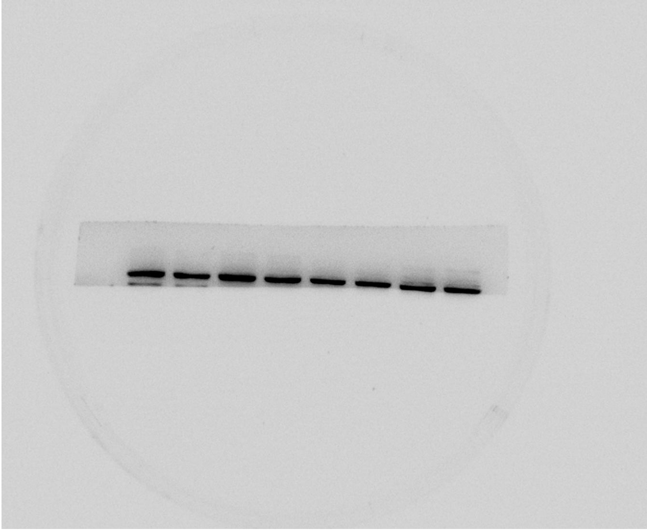

GAPDH

Figure S2L

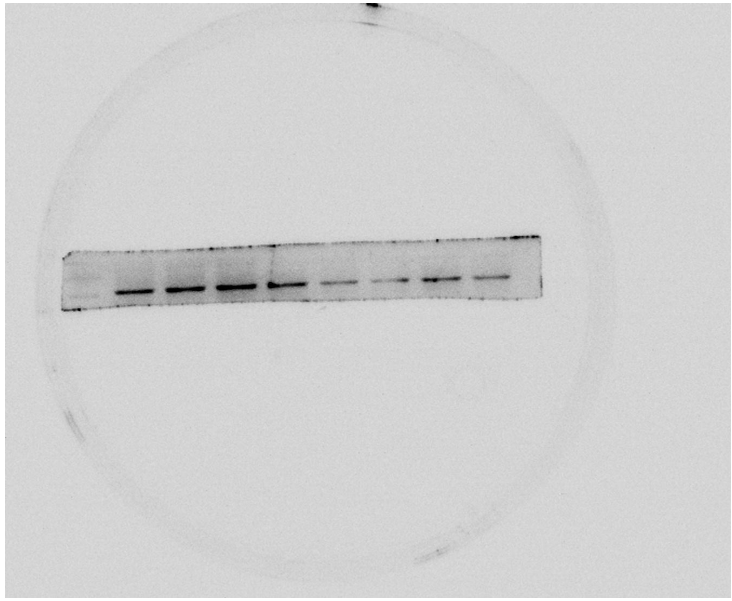

SLC7A11

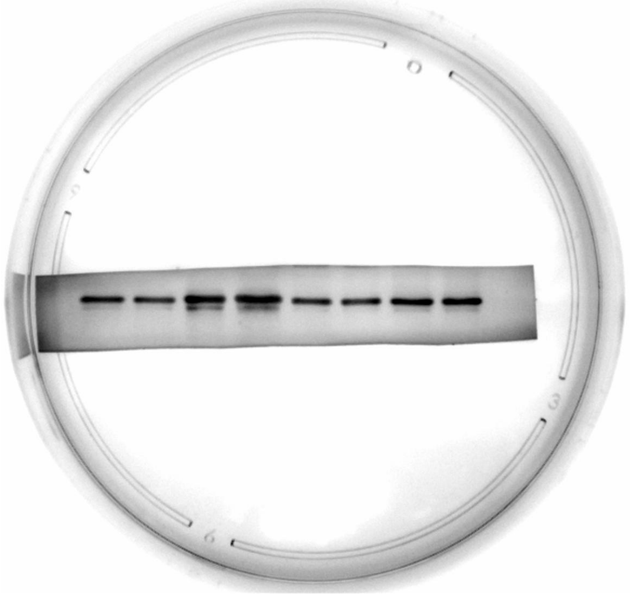

GPX4

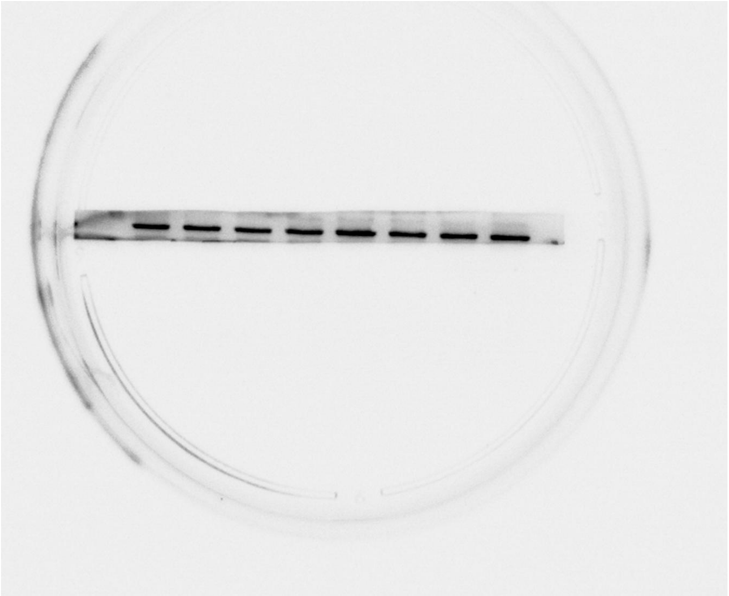

GAPDH

Figure S3A

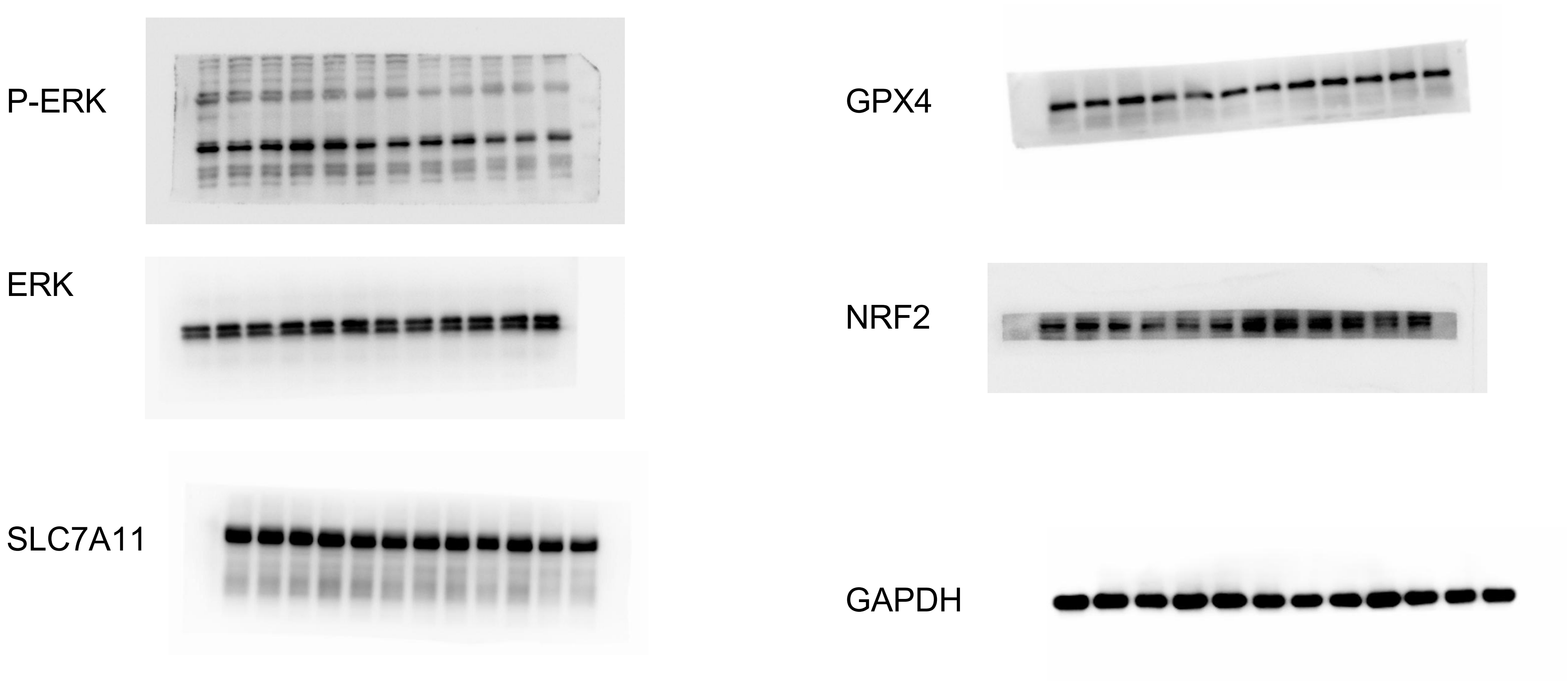

Supplement: Supplementary file 3 — Full and uncropped western blots [file 41420_2025_2331_MOESM3_ESM.pdf]
